# An inhibitor of chondroitin sulfate proteoglycan synthesis promotes central nervous system remyelination

**DOI:** 10.1038/ncomms11312

**Published:** 2016-04-26

**Authors:** Michael B. Keough, James A. Rogers, Ping Zhang, Samuel K. Jensen, Erin L. Stephenson, Tieyu Chen, Mitchel G. Hurlbert, Lorraine W. Lau, Khalil S. Rawji, Jason R. Plemel, Marcus Koch, Chang-Chun Ling, V. Wee Yong

**Affiliations:** 1 Department of Clinical Neurosciences, Hotchkiss Brain Institute, University of Calgary, 3330 Hospital Drive NW, Calgary, Alberta, Canada T2N 4N1; 2 Department of Chemistry, University of Calgary, 2500 University Drive NW, Calgary, Alberta, Canada T2N 1N4

## Abstract

Remyelination is the generation of new myelin sheaths after injury facilitated by processes of differentiating oligodendrocyte precursor cells (OPCs). Although this repair phenomenon occurs in lesions of multiple sclerosis patients, many lesions fail to completely remyelinate. A number of factors have been identified that contribute to remyelination failure, including the upregulated chondroitin sulfate proteoglycans (CSPGs) that comprise part of the astrogliotic scar. We show that *in vitro*, OPCs have dramatically reduced process outgrowth in the presence of CSPGs, and a medication library that includes a number of recently reported OPC differentiation drugs failed to rescue this inhibitory phenotype on CSPGs. We introduce a novel CSPG synthesis inhibitor to reduce CSPG content and find rescued process outgrowth from OPCs *in vitro* and accelerated remyelination following focal demyelination in mice. Preventing CSPG deposition into the lesion microenvironment may be a useful strategy to promote repair in multiple sclerosis and other neurological disorders.

## Abstract (web summary)

Chondroitin sulfate proteoglycans (CSPGs) secreted by astrocytes are known to inhibit OPC differentiation and remyelination. Here, the authors identify a novel CSPG synthesis inhibitor and find it can rescue OPC differentiation *in vitro* and accelerate remyelination in mice following focal demyelination.

## Graphical abstract

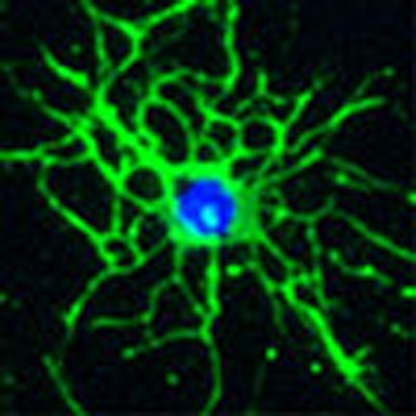

Multiple sclerosis (MS) is a chronic demyelinating disease of the central nervous system (CNS), characterized by immune cell infiltration, demyelination and neuroaxonal damage. Remyelination is the generation of new myelin sheaths after injury; it occurs spontaneously in animal demyelinating models and human disease but the majority of lesions in MS patients fail to completely remyelinate^1^,^2^,^3^. In addition to providing rapid saltatory conduction of action potentials, myelin provides axons a source of metabolites for their general wellbeing^4^,^5^; indeed, chronic demyelinated axons are more prone to irreversible damage and loss^6^. It is therefore necessary to develop strategies to promote remyelination when it fails in MS and other demyelinating disorders^7^.

For remyelination to occur, oligodendrocyte precursor cells (OPCs), which are situated throughout the adult CNS and are capable of making and remodelling myelin throughout the lifespan^8^,^9^, must first proliferate and migrate into demyelinating lesions; subsequent maturation into oligodendrocytes, involving the extension of processes that contact, enwrap and compact around axons complete the remyelination program. The cause of remyelination failure is likely several-fold and includes lesion-associated factors that impede any of the aforementioned steps^10^. These inhibitory cues include Wnt and Notch signalling pathways^11^,^12^, semaphorins^13^,^14^, Lingo-1 (ref. 15), myelin debris^16^, as well as extracellular matrix (ECM) molecules such as hyaluronan and chondroitin sulfate proteoglycans (CSPGs)^17^,^18^,^19^.

CSPGs are a family of large molecules consisting of a single protein backbone tethered to few or many repeating disaccharide (glucuronic acid and *N*-acetyl-galactosamine) polymers called chondroitin sulfate. Further heterogeneity of CSPG structure results from several sulfation sites by different enzymes on each disaccharide pair. CSPGs, which act as guidance and signalling molecules during development, maintain the structural integrity of the healthy CNS in specialized structures such as basement membranes, perineuronal nets and nodes of Ranvier^20^. In the damaged CNS, CSPGs become highly upregulated as part of the astrogliotic scar, and are potent inhibitors of axon regeneration after traumatic injury^20^,^21^. Several approaches to neutralize CSPGs after injury have included digestion of CSPGs with the enzyme chondroitinase ABC^22^,^23^, RNA interference of chain polymerization enzymes^24^, and peptide blocking of protein tyrosine phosphatase sigma^25^, one of the recently characterized CSPG receptors^26^.

A number of CSPG members are upregulated in MS lesions, including versican, aggrecan and neurocan^27^. We and others have previously shown that CSPGs inhibit morphological differentiation of OPCs *in vitro* and remyelination *in vivo*^19^,^28^. In this report, we attempt two strategies to overcome the inhibitory nature of CSPGs on OPC growth: first, to screen for drugs that permit OPCs to grow in the presence of inhibitory CSPGs, and second, to test the efficacy of a small molecule CSPG synthesis inhibitor^29^ in novel applications to the CNS. Our results emphasize the difficulty of overcoming CSPGs once deposited into the extracellular environment, and we demonstrate that the reduction of CSPG production after demyelination *in vivo* enhances remyelination.

## Results

### CSPGs inhibit oligodendrocyte growth in culture

**Figure 1:**
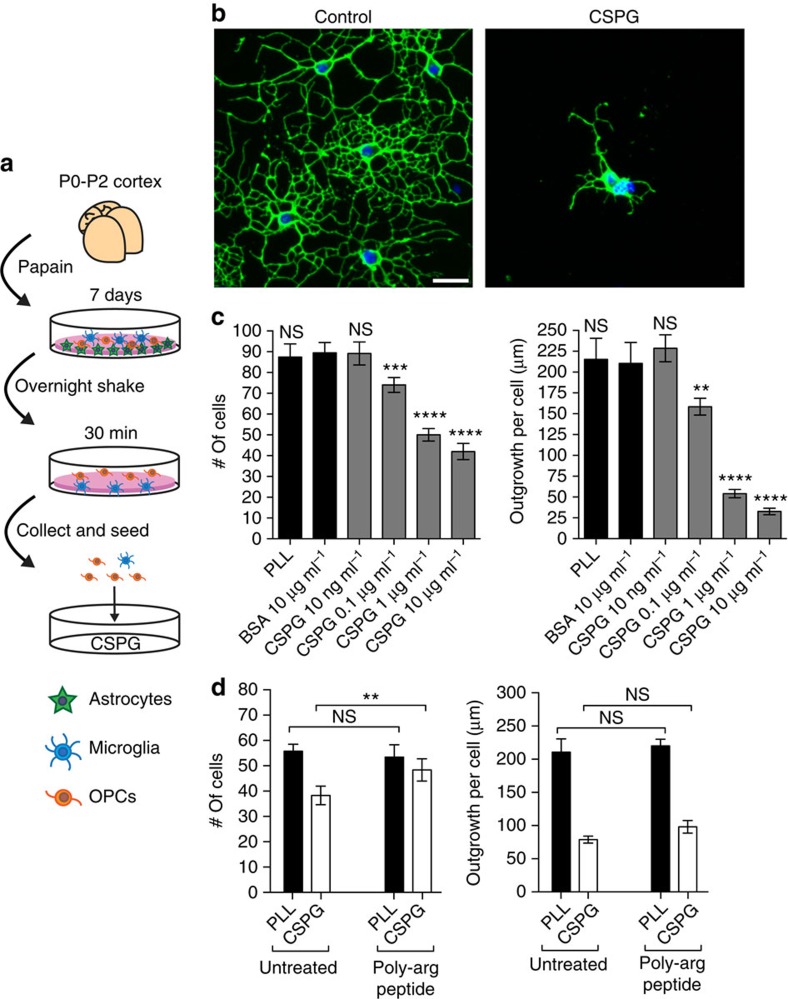
OPCs are inhibited by CSPGs *in vitro*. (**a**) Schematic of enrichment of OPCs from mixed glial cultures and their seeding onto commercial CSPGs. (**b**) Representative images of OPCs stained for the sulfatide O4 18–24 h after plating onto bovine serum albumin (BSA, control substrate) or CSPGs. (**c**) OPCs plated onto CSPGs have reduced adherence as well as process outgrowth in a concentration-dependent manner. **P<0.01, ***P<0.001, ****P<0.0001, one-way analysis of variance (ANOVA) with Dunnett's *post hoc* test (respective of BSA control). PLL, poly-L-lysine. (**d**) Addition of a poly-arginine peptide rescues the CSPG inhibitory effect of OPC adhesion but not process outgrowth. **P<0.01, two-way ANOVA with Sidak's multiple comparisons. Results are presented as four replicate wells of individual experiments that were conducted three times. Error bars are mean±s.d. Scale bar, 25 μm.

First, we confirmed that a commercial mixture of CSPGs (containing mostly neurocan, phosphacan, versican and aggrecan) inhibited the growth of oligodendrocytes *in vitro*. Murine OPCs were enriched from mixed glial cultures through a series of differential adhesion steps (Fig. 1a). This procedure resulted in cultures of >75% purity as detected by the intermediate oligodendrocyte marker O4 (Supplementary Fig. 1), with the major contaminant being ionized calcium-binding adapter molecule 1 (Iba1)+ microglia. OPCs were seeded onto 96-well plates coated with CSPGs (10 ng ml^−1^–10 μg ml^−1^) for 18–24 h, followed by staining for O4 (see Supplementary Table 1 for antibodies used). OPCs plated onto a control substrate of bovine serum albumin (BSA) displayed elaborate arborization of processes (Fig. 1b), which can be viewed as a correlate of myelination potential since the terminal end of each process is capable of producing a myelin sheath. In contrast, OPCs plated onto CSPGs displayed significantly reduced cell adhesion and process outgrowth in a concentration-dependent manner (Fig. 1b,c). To address whether the negative charges on chondroitin sulfate are responsible for reduced adhesion and process outgrowth, we treated OPCs plated onto CSPGs with a positively charged 12 amino acid poly-arginine peptide to counteract the negative charges. We found that while the poly-arginine peptide rescued the reduced adhesion of OPCs on CSPGs, it did not reverse the reduced process outgrowth (Fig. 1d). Thus, we focused our attention on process outgrowth as a more biologically relevant phenomenon for inhibition by CSPGs, since it was not due to ionic charge.

### Screening drugs to promote growth in the presence of CSPGs

**Figure 2:**
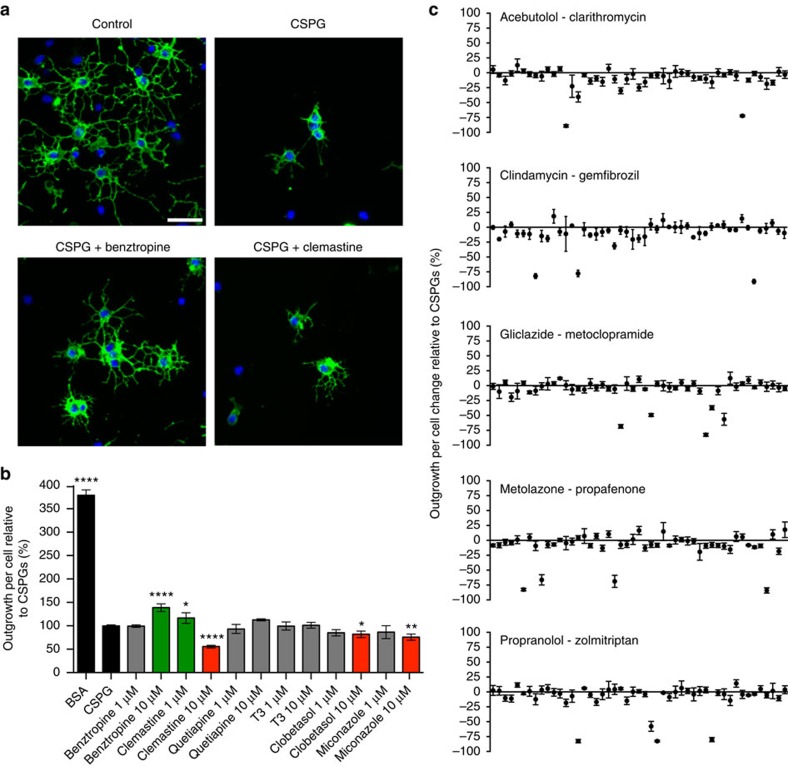
A screen of existing medications have minimal effects on rescuing OPC growth on CSPGs. (**a**) Representative images of OPCs plated onto either BSA (control), CSPGs or CSPGs in the presence of benztropine (10 μM) or clemastine (1 μM) for 18–24 h, stained with O4. (**b**) Quantifications of process outgrowth of OPCs plated on CSPGs in the presence of a number of reported pro-remyelinating compounds, demonstrating a small effect with benztropine (10 μM) and clemastine (1 μM), while showing toxicity with higher concentrations of clemastine, clobetasol and miconazole (10 μM). (**c**) Process outgrowth of OPCs plated on CSPGs treated with a screen of 245 drugs, presented as change relative to CSPGs. None of these compounds were statistically significant for positive change compared with CSPGs, while some compounds were toxic (negative change; see Supplementary Table 3). The results are presented as four replicate wells of an individual experiment that was conducted three times (**b**) or once (**c**). *P<0.05, **P<0.01, ****P<0.0001, one-way analysis of variance with Dunnett's *post hoc* test (respective to CSPG control). Error bars are mean±s.d. Scale bar, 25 μm.

Given that CSPGs are present in demyelinating lesions^19^,^27^, we next sought to find drug candidates to promote OPC growth in the presence of inhibitory CSPGs. We treated OPCs with different drugs (1 or 10 μM) immediately after plating onto 1 μg ml^−1^ CSPGs, and measured morphology 18–24 h later (Fig. 2a). Recent screens identified benztropine, clemastine, quetiapine, clobetasol and miconazole as agents which promote OPC differentiation *in vitro* and remyelination *in vivo*^30^,^31^,^32^. Benztropine had no rescued effect at 1 μM, and a small but statistically significant increase in process outgrowth relative to CSPGs at 10 μM (Fig. 2b). Clemastine showed an even smaller significant rescued phenotype at 1 μM and was toxic to OPCs at 10 μM. Quetiapine, clobetasol, miconazole, as well as thyroid hormone (T3) that is a positive control for OPC differentiation^30^, showed no increase in process outgrowth at either concentration; clobetasol and miconazole were mildly toxic at 10 μM. Thus, although others have reported an enhancement in OPC differentiation under control conditions with these medicines, we find that the presence of lesional factors such as CSPGs prevents such improvement.

Next, we tested a subset of drugs from the National Institute of Neurological Disorders and Stroke Custom Collection 2 drug library of 1,040 compounds^33^. A total of 245 drugs were chosen that had known oral bioavailability, likely CNS penetrating capacity, regulatory approval by Health Canada, and that covered a broad range of drug classes (Supplementary Table 2). At a concentration of 10 μM, none of the 245 compounds showed significant rescue of process outgrowth compared with CSPGs, and a portion of the compounds were toxic (Fig. 2c and Supplementary Table 3). We were, therefore, unable to find any candidate agents to overcome CSPG inhibition of OPCs.

### Fluorosamine reduces the synthesis of CSPGs by astrocytes

**Figure 3:**
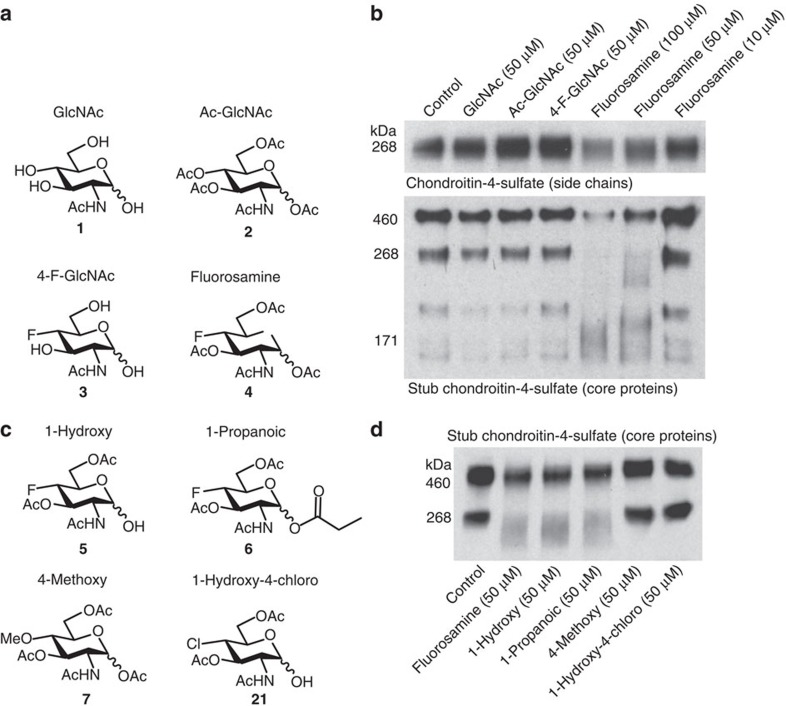
Fluorosamine reduces the synthesis of CSPGs by astrocytes. (**a**) Chemical structures for GlcNAc and its derivatives. (**b**) Western blots for chondroitin-4-sulfate (sidechains) and stub chondroitin-4-sulfate (core proteins) show a concentration-dependent decrease in total CSPG content with fluorosamine treatment. (**c**) Chemical structures of four derivatives of fluorosamine with various substitutions to carbon-1 and carbon-4. (**d**) The 1-hydroxy and 1-propanoic fluorosamine derivatives were effective at reducing CSPG synthesis, whereas the 4-methoxy and 1-hydroxy-4-chloro compounds were ineffective. The results are presented as an individual experiment that was conducted three times.

Given the limited success of our drug screen, we rationalized that a better approach to overcome CSPG inhibition of OPCs would be to reduce their synthesis in gliotic scars. It is known from the axonal regeneration literature that a significant portion of the inhibitory properties of CSPGs derive from their chondroitin sulfate side chains^22^,^23^. Chondroitin sulfate is a polysaccharide that is synthesized by a number of glycosyltransferase enzymes using the sugars glucuronic acid and *N*-acetyl-galactosamine following a brief linker region between the chain and the protein core (Supplementary Fig. 2). UDP-*N*-acetyl-galactosamine is converted from UDP-*N*-acetyl-glucosamine (GlcNAc, **1**, Fig. 3a) by the enzyme 4-epimerase. A newly characterized fluorinated analogue of GlcNAc has been shown to reduce the content of chondroitin sulfate side chains in cultured smooth muscle cells, most likely by interfering with the enzymatic conversion of naturally occurring UDP-GlcNAc to UDP-*N*-acetyl-galactosamine by 4-epimerase^29^. 4-F-GlcNAc (**3**, Fig. 3a) can be acetylated on its three terminal hydroxyl groups (→**4**, Fig. 3a) to increase penetrance across the cell membranes. Once entered into the cytoplasm, the acetate groups are hydrolysed by esterases, releasing the 4-F-GlcNAc (**3**), which could be ultimately converted to the 4-epimerase inhibitor: UDP-4-F-GlcNAc. We therefore tested whether peracetylated 4-F-GlcNAc (**4**, henceforth referred to as fluorosamine for brevity) could reduce synthesis of chondroitin sulfate on CSPGs from cultured astrocytes. We synthesized fluorosamine by following a modified route from *N*-acetyl-glucosamine (Supplementary Fig. 3, Supplementary Methods).

Cultured astrocytes secrete CSPGs into their media, particularly upon stimulation with cytokines such as transforming growth factor (TGF)-β1 (ref. 34). We detected both intact chondroitin sulfate sidechains with the 2H6 antibody, and core proteins using the BE-123 antibody against the stubs of chondroitin sulfate following chondroitinase ABC pre-digestion, finding diffuse staining of bands (Fig. 3b); others have reported those bands >200 kDa as a heterogeneous population of large CSPGs^34^,^35^. The treatment with GlcNAc (**1**), acetylated GlcNAc (**2**) and 4-F-GlcNAc (**3**) did not alter the level of CSPGs (Fig. 3b). However, fluorosamine (**4**), with peracetylation and the 4-F moiety, reduced both the amount of chondroitin sulfate side chains as well as CSPG core proteins in the media. We also confirmed that fluorosamine reduced CSPG synthesis in cultured primary human astrocytes (Supplementary Fig. 4a) without causing toxicity (Supplementary Fig. 4b). We found that a ratio of at least 10:1 was required for fluorosamine compared with acetylated GlcNAc to inhibit astrocyte synthesis of CSPGs (Supplementary Fig. 5), suggesting that fluorosamine acts as an alternative substrate to naturally occurring GlcNAc.

To determine the structural activity of fluorosamine, we synthesized a number of derivatives with substitutions to various carbon positions (**5**–**7**, **21**, Fig. 3c and Supplementary Fig. 6). Substitutions of a hydroxyl group (**5**) or propanoate ester (**6**) to carbon-1 of fluorosamine did not change its efficacy to reduce CSPG synthesis, while replacing the fluorine with a methoxy (**7**) or chlorine (**21**) on carbon-4 alongside a hydroxyl on carbon-1 ablated the effect. The methoxy and chloride groups may sterically hinder the molecule's ability to fit in the binding site of the 4-epimerase enzyme, reinforcing the necessity of a fluorine atom in the carbon-4 position to reduce CSPG synthesis (Fig. 3d).

### Fluorosamine-treated astrocyte matrix is permissive for OPCs

**Figure 4:**
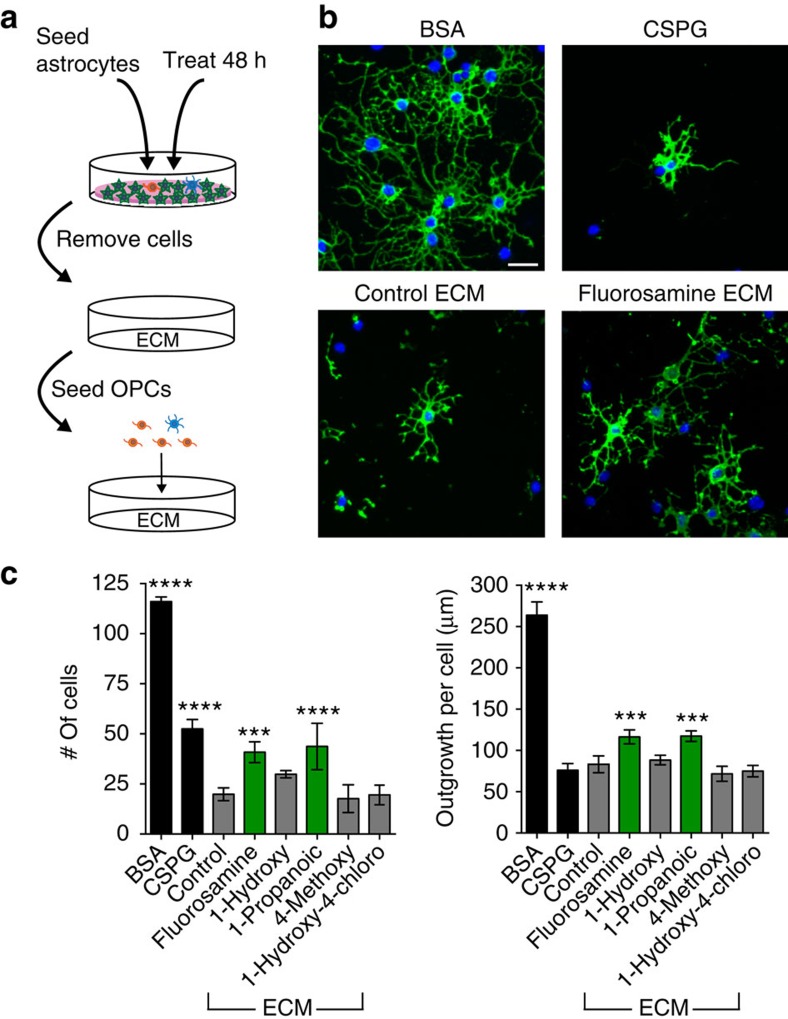
Fluorosamine-treated astrocyte matrix is less inhibitory for OPC growth. (**a**) Schematic of treated astrocytes that are removed from the plate bottom, leaving behind an anchored matrix which OPCs are seeded upon. (**b**) Representative images of OPCs plated onto either BSA, CSPGs, untreated control astrocyte ECM or fluorosamine treated astrocyte ECM, where they have greater adherence and outgrowth. (**c**) Quantification of cell number and process outgrowth, showing a partially rescued phenotype with fluorosamine and its 1-propanoic derivative treatment, and no effect with the 1-hydroxy, 4-methoxy or 1-hydroxy-4-chloro compounds. The results are presented as four replicate wells of an individual experiment that was conducted two times. ***P<0.001, ****P<0.0001 compared with astrocyte ECM control; one-way analysis of variance with Dunnett's *post hoc* test. Error bars are mean±s.d. Scale bar, 25 μm.

We next tested the effect of reducing astrocyte CSPG synthesis on OPCs in culture. We seeded astrocytes in 96-well plates in the immediate presence of fluorosamine to alter the CSPG proportion of a generalized ECM that is secreted and adhered to the plate bottom (Fig. 4a)^36^. To exclude any cell–cell contact effects between astrocytes and OPCs, astrocytes were removed from the plate bottom 7 days after treatment using 0.2 g l^−1^ EDTA^36^. OPCs were then plated on the ECM left behind for 18–24 h and process outgrowth was compared with 1 μg ml^−1^ BSA and commercial CSPGs. We found that OPCs plated onto control astrocyte matrix were even more inhibited than with commercial CSPGs (Fig. 4c). However, OPCs plated onto fluorosamine or the 1-propanoic fluorosamine-derivative-treated astrocyte matrix had greater cell adhesion as well as process outgrowth, whereas the 4-methoxy and 1-hydroxy-4-chloro analogues had no effect (Fig. 4b,c); the 1-hydroxy fluorosamine analogue also did not overcome the inhibitory effect of the astrocyte ECM. These results confirm that fluorosamine treatment creates a more permissive environment for OPC growth.

### Demyelination-induced CSPGs are mitigated with fluorosamine

**Figure 5:**
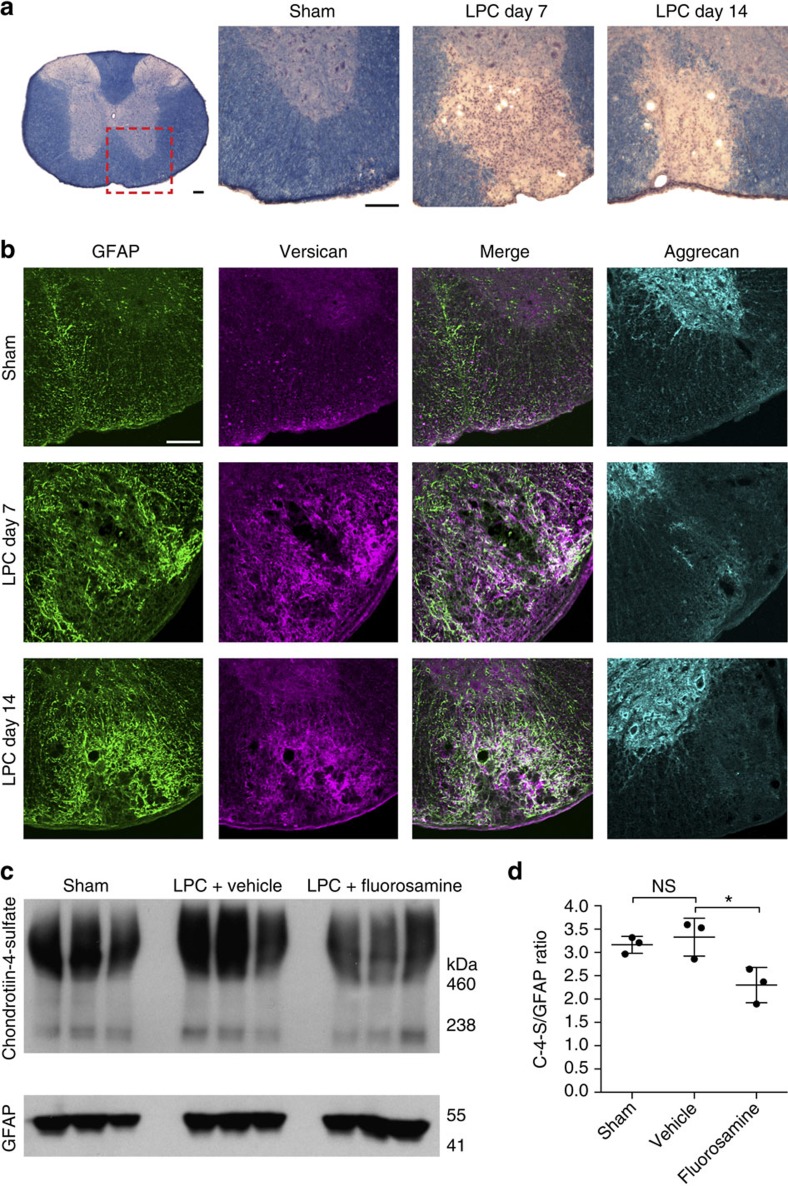
Fluorosamine reduces CSPG deposition following lysolecithin demyelination. (**a**) Representative eriochrome cyanine-stained sections of spinal cords after lysolecithin demyelination. Red outlined box represents location of images in the subsequent panels. (**b**) Representative immunohistochemistry images of GFAP, versican and aggrecan show persistent versican accumulation at the lesion site that correlates with reactive astrogliosis. Aggrecan, which is found in abundance in the grey matter, presumably in perineuronal nets (observed as rings of staining), is absent in the area of lysolecithin demyelination. (**c**) Western blots of spinal cords 7 days after lysolecithin and fluorosamine treatment show reduced abundance of chondroitin-4-sulfate despite comparable GFAP intensity. (**d**) Quantifications show a decreased intensity of chondroitin-4-sulfate relative to GFAP intensity in fluorosamine compared with vehicle-treated animals. The results are presented as representative images of five animals per group (**b**) or as three animals per group in individual lanes (**c**). *P<0.05, one-way analysis of variance with Tukey's *post hoc* test. Error bars are mean±s.d. Scale bar, 100 μm.

We previously reported that CSPGs, detected using the side chain antibody CS-56, were increased following injection of the detergent lysolecithin into the spinal cord white matter of mice^19^. Here we followed up this finding to ask which particular CSPG family members found in MS lesions^27^ were upregulated at demyelinating (7 days) and remyelinating (14 days) time points (Fig. 5a)^12^,^19^,^37^. Compared with saline-injected sham animals, 0.5 μl of 1% lysolecithin injected into the ventral spinal cord white matter resulted in a pronounced astrocytic response detected by elevation of glial fibrillary acidic protein (GFAP) that persisted over 14 days (Fig. 5b). Using a combination of antibodies directed to the major isoforms of versican^38^, we found an increase in fluorescence intensity at the lesion site, closely associated with areas of GFAP intensity. In contrast, we found no alteration of aggrecan, which was confined to the grey matter of the spinal cord.

We next tested whether fluorosamine reduces the content of CSPGs in demyelinated lesions. Lysolecithin-inflicted mice were treated from 3 to 7 days post surgery with fluorosamine (200 mg kg^−1^ intraperitoneally daily) and killed at 7 days. A 2 mm coronal piece of tissue, estimated to comprise the centre of the ventral lesion but also non-demyelinated dorsal and lateral column tissues, was lysed and processed for western blot. There was a significant decrease of chondroitin sulfate content normalized to GFAP in fluorosamine compared with vehicle animals (Fig. 5c,d), confirming that fluorosamine reduces the CSPG burden following lysolecithin demyelination.

### Fluorosamine treatment promotes remyelination

**Figure 6:**
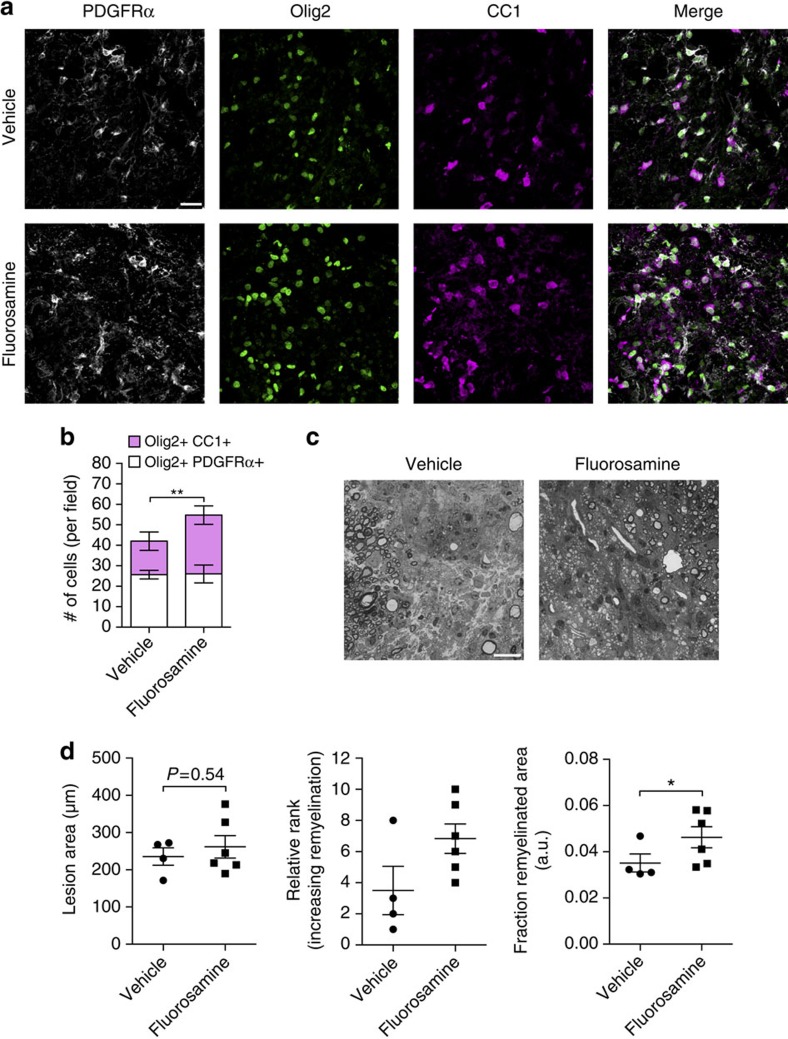
Fluorosamine promotes remyelination *in vivo*. (**a**) Representative immunohistochemistry images of PDGFRα, Olig2 and CC1 in the core of lysolecithin lesions 7 days after injury. (**b**) Quantifications show a significant increase in the number of Olig2+CC1+ mature oligodendrocytes in fluorosamine-treated animals, with no difference in Olig2+PDGFRα+ OPCs. (**c**) Representative toluidine blue-stained semithin sections of lysolecithin lesions 14 days after injury. (**d**) Quantification shows a significant greater area occupied by remyelinated sheaths in fluorosamine compared with vehicle-treated animals that agreed with blinded rank order analysis, with no difference between total lesion area. The results are presented from four to six animals per group. a.u., arbitrary units. *P<0.05, Mann–Whitney *U*-test; **P<0.01, Student's *t*-test. Error bars are mean±s.d. (**b**) or s.e.m. (**d**). Scale bar, 25 μm.

Next, we tested whether fluorosamine can accelerate remyelination. The mice were treated with fluorosamine (50 mg kg^−1^ intraperitoneally daily) or saline vehicle starting 3 days post surgery. At 7 days, a subset of animals were killed and tissue processed for immunohistochemistry. The cells of the oligodendrocyte lineage were probed with a triple stain for platelet-derived growth factor receptor (PDGFR)α (for OPCs), CC1 (for mature oligodendrocytes) and Olig2 (for all oligodendrocyte lineage cells) at the core of the lesions (Fig. 6a). Although the number of Olig2+PDGFRα+ OPCs was comparable between groups, fluorosamine-treated animals had significantly more Olig2+CC1+ mature oligodendrocytes compared with vehicle-treated animals (Fig. 6b).

Another set of animals were injected daily from 3 days post surgery until 14 days, where they were killed and tissue processed for semi-thin sections to examine fine myelin architecture (Supplementary Fig. 7). Remyelination capacity was calculated as a fraction of the lesion area occupied by remyelinated sheaths. There was a significantly higher fraction of remyelinated area in the fluorosamine-treated animals compared with vehicle controls, with no difference in total lesion area (Fig. 6c,d). Overall, these experiments showed that fluorosamine treatment accelerates the rate of oligodendrocyte maturation and remyelination *in vivo*.

### Fluorosamine treats inflammatory demyelination

**Figure 7:**
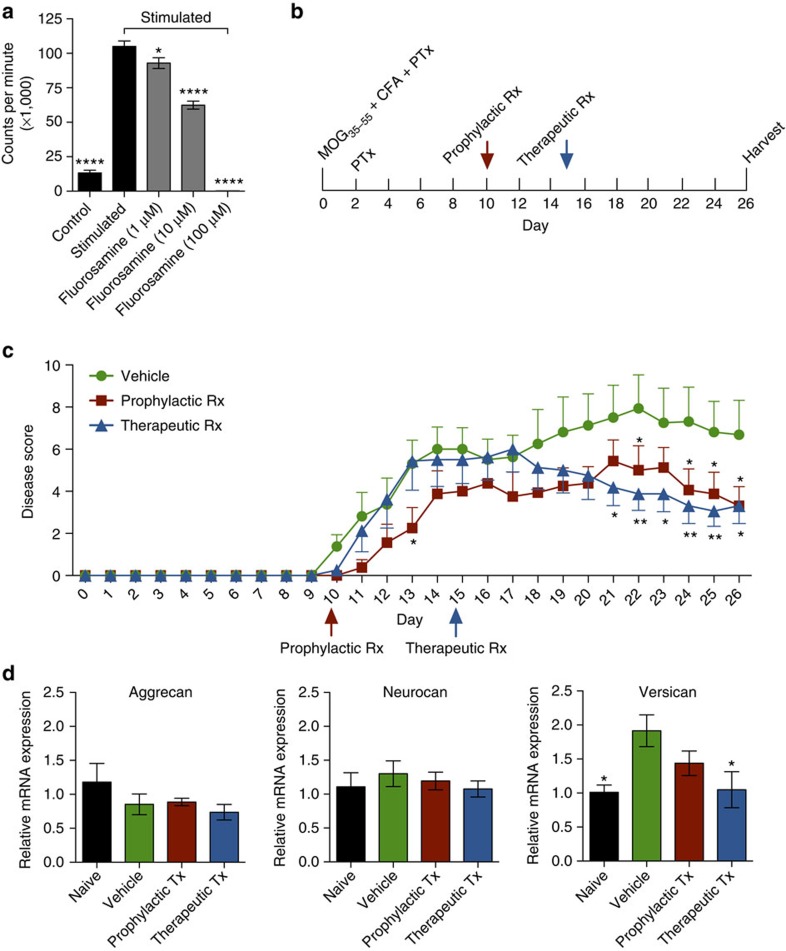
Fluorosamine reduces the proliferation of T cells and ameliorates EAE. (**a**) Proliferation of splenocytes activated with anti-CD3 and anti-CD28 antibodies is significantly reduced in a concentration-dependent manner by fluorosamine. (**b**) Schematic for the experimental EAE paradigm and treatment starting points. (**c**) Disease scores of EAE animals over the course of a 26-day experiment. The animals were treated either prophylactically at day 10 or therapeutically at day 15, compared with vehicle treatment. Both the treatment groups had reduced disease scores on the last few days of the experiment. (**d**) Isolated RNA from the spinal cords of EAE mice harvested at day 26 shows significant increases in versican in vehicle-treated EAE animals compared with naive subjects, and this is decreased with therapeutic fluorosamine treatment. Results are presented as four replicate wells of an individual experiment conducted twice (**a**), or from seven to eight animals per group (**c**,**d**). MOG, myelin oligodendrocyte glycoprotein; CFA, Complete Freund's Adjuvant; PTx, pertussis toxin. *P<0.05, **P<0.01, ****P<0.0001 one-way analysis of variance (ANOVA) with Dunnett's *post hoc* test (**a**,**d**) or two-way repeated-measures ANOVA with Holm–Sidak's *post hoc* test (**c**). Error bars are mean±s.e.m.

All currently approved medications for multiple sclerosis have strong immunomodulatory properties and have been developed for this mechanism of action. We therefore assessed whether fluorosamine also has immunomodulatory properties. We first tested the effects of fluorosamine on splenocytes isolated in culture; T lymphocytes were activated with anti-CD3 and anti-CD28 antibodies in the presence of fluorosamine for 48 h, and proliferation was determined by the uptake of tritiated thymidine. Fluorosamine reduced proliferation in a concentration-dependent manner (Fig. 7a), suggesting that fluorosamine confers immunomodulatory properties. We next tested fluorosamine treatment in the characteristic inflammatory model of multiple sclerosis, experimental autoimmune encephalomyelitis (EAE). The mice were immunized in a standard protocol with myelin oligodendrocyte glycoprotein peptide and associated adjuvants (Fig. 7b). Once symptomatic at day 10, a subset of mice were treated with fluorosamine (50 mg kg^−1^ intraperitoneally daily) or vehicle (prophylactic treatment); another group of animals were treated with fluorosamine beginning at day 15 when mice had significant clinical disability (therapeutic treatment). Both groups had significantly lower disease scores compared with vehicle treatment for the last few days of the experiment (Fig. 7c). The animals were subsequently killed and spinal tissue processed for RNA quantification. We performed PCR for different CSPG members and found that versican was significantly upregulated in vehicle-treated EAE animals and reduced with therapeutic fluorosamine treatment (Fig. 7d); both aggrecan and neurocan were unchanged in EAE. In summary, fluorosamine promotes behavioural recovery in EAE, correspondent with a reduction of versican transcripts in the spinal cord.

## Discussion

We sought first to identify approved medicines that could rescue the inhibitory phenotype caused by CSPGs. This was generally an unsuccessful approach, and emphasized that even drugs found recently to promote the maturation of oligodendrocytes in the absence of inhibitory stimuli (benztropine, clemastine, quetiapine, clobetasol and miconazole)^30^,^31^,^32^ could not effectively overcome the CSPG barrier. From this result, we rationalized that inhibitors of CSPG production would constitute a rational approach to therapy. It is likely that drugs not included in our relatively small screen could reverse the inhibition of CSPGs on OPCs; a more thorough understanding of both the CSPG receptors expressed by OPCs, as well as downstream signalling cascades, will direct future therapeutic targets. Combinational approaches of both neutralizing the inhibitory environment as well as promoting differentiation of OPCs should be more effective than either strategy alone.

Impressive results have been reported from the use of the bacterial enzyme chondroitinase ABC following traumatic injury, either alone or in combination with an adjunctive therapy such as growth factors or task-specific rehabilitation to encourage axonal regrowth and innervation or plasticity of spared circuits^39^. Given that chondroitinase ABC must be administered via local injection into injury sites, we deemed it impractical for demyelinating diseases such as MS where lesions arise all throughout the brain and spinal cord. Instead, we opted to use a systemic agent that could potentially target CSPG deposition in any evolving lesion. This study is, to the best of our knowledge, the first report of the use of a glucosamine analogue as an inhibitor of CSPG synthesis in the nervous system. We first found that fluorosamine reduced the content of chondroitin sulfate and CSPG core proteins from cultured astrocytes, probably by inhibiting the conversion of UDP-*N*-acetyl-glucosamine to UDP-*N*-acetyl-galactosamine by the enzyme 4-epimerase^29^,^40^. Indeed, a ratio of 10:1 fluorosamine to acetylated GlcNAc was required to observe a decrease in CSPG synthesis, suggesting that fluorosamine acts as an alternative substrate for GlcNAc and not as a traditional competitive inhibitor. Fluorosamine reduced the chondroitin sulfate content of astrocyte-conditioned media, and it appeared to also mitigate the export of full-length core proteins. It is possible that the mechanisms of export require the presence of intact side chains as a regulatory checkpoint to prevent improperly constructed CSPGs from being secreted.

We previously showed that astrocytes in culture produce an ECM rich with CSPGs that is inhibitory for neurite outgrowth, and this inhibition can be partly overcome by digestion with chondroitinase ABC and other agents^36^. In this report, we found that immediately treating seeded astrocytes with fluorosamine reduces the inhibitory nature of this ECM on oligodendrocyte process extension without being toxic to astrocytes. The finding that the astrocyte ECM was more inhibitory than commercial CSPGs alone could be due to an increased concentration of CSPGs in the astrocyte ECM compared with commercial CSPGs, as we showed a concentration-dependent effect of CSPGs on process extension, and although we quantified a robust decrease in CSPGs in the conditioned media, this may not have been the case for the deposited ECM. Alternatively, the relative amounts of CSPG constituents are likely different between astrocyte ECM and commercial CSPGs, and the relative inhibitory properties of individual CSPGs on oligodendrocyte growth is presently unknown. It is also likely that there are other, presently undefined factors in astrocyte ECM that exert potent inhibitory action on oligodendrocyte process growth; if fluorosamine selectively reduces CSPG secretion but not these other factors, the capacity of a rescued phenotype would be limited. Since astrocytes are also known to produce factors that enhance oligodendrocyte process outgrowth, such as basic fibroblast growth factor^41^, it is important that only the inhibitory components of the astrocyte secretome be targeted therapeutically.

We demonstrated that fluorosamine has dual modalities by inhibiting CSPG synthesis as well as being immunomodulatory. It is possible that fluorosamine affected CSPGs at the RNA level by creating an anti-inflammatory environment with less-reactive astrocytes in the EAE-inflicted spinal cord. The immunomodulatory action of fluorosamine in EAE may be due to similar mechanisms of the base molecule, *N*-acetylglucosamine, which inhibits T cells and treats EAE by promoting N-branching^42^; alternatively, it could be related to decreased leukocyte migration by downregulation of selectin ligands^43^. Future work will also need to determine the relative contribution of immunomodulation and remyelination in promoting behavioural recovery from EAE.

There still remain many unanswered questions about the relationship between CSPGs and cells of the oligodendrocyte lineage. First, the role of individual CSPGs on oligodendrocyte behaviour needs to be addressed. MS lesions are rich with versican, aggrecan, neurocan and others, and the commercial CSPG mixture containing these species is potently inhibitory for process extension. However, lysolecithin results in only an observable change in versican, which may have a minor role in inhibiting OPCs given that remyelination is an endogenous response that is efficient in young animals. Versican is further complicated by the fact that it has four mRNA splice variant isoforms, and there are conflicting reports if all of them have inhibitory functions, with one report actually suggesting a growth promoting effect of versican V1 (ref. 44). There have now been described many lesion associated factors that are inhibitors of the remyelination response, including cell intrinsic factors such as Notch and Wnt pathways^11^,^12^, and extrinsic factors such as myelin debris^16^, Lingo-1 (ref. 15), semaphorins^13^,^14^, hyaluronan^17^,^18^ and CSPGs. The relative contribution of each of these inhibitory factors deserves evaluation. One may argue that the best chance to promote remyelination would be to stimulate macrophages and microglia to collectively clear the inhibitory microenvironment; after all, OPCs appear not to lose their migration and differentiation capacity even in aged animals, but are constrained by an ongoing immunosenescence in macrophages and microglia to clear debris^45^. Whatever the case, it is clear that the growing collection of remyelination inhibitors cannot be overlooked. Herein, our results have yielded one candidate approach to reduce the synthesis of inhibitory CSPGs for repair of the CNS following a demyelinating injury. As CSPGs are also impediments to axonal regrowth, fluorosamine should also have favourable considerations to enable axonal regeneration.

## Methods

### Mixed glial cultures

All murine *in vitro* experiments were performed in accordance with ethical animal care guidelines by the Animal Care Committee at the University of Calgary. Mixed glial cultures were generated from the brains of neonatal CD-1 mice^46^,^47^. Post-natal day (P)0–P2 pups (10–12 per litter) of mixed gender were killed by decapitation, and the brains were removed from the skull and placed in Hank's Balanced Salt Solution (HBSS) on ice. Both cortices were isolated from the telencephalon, and overlying meninges were removed with fine forceps. Cortices from all the mice were pooled and minced with a scalpel to approximately 1 mm^3^ pieces. The tissue was collected in a 50 ml centrifuge tube containing 350 μl HBSS per brain.

The tissue was mechanically dissociated with a 1 ml micropipettor and warmed to 37 °C. A digestion solution was prepared containing 1.54 mg ml^−1^ papain, 360 μg ml^−1^ L-cysteine and 60 mg ml^−1^ DNAseI. Seventy-five microlitres per brain of the digestion solution was added to the dissociated tissue for 30 min at 37 °C. Following digestion, the cells were triturated with a 1 ml micropipettor and the reaction was stopped by filling the centrifuge tube with mixed glial media (MGM, Dulbecco's Modified Eagle Media (DMEM) containing 10% heat-inactivated fetal bovine serum, 1% L-glutamine, 1% sodium pyruvate and 1% penicillin/streptomycin). The single cell suspension was centrifuged at 1,200 r.p.m. for 10 min. After decanting the media, the pellet was resuspended with 3 ml MGM and divided into 3 T-75 poly-L-lysine-coated tissue culture flasks containing 9 ml MGM. The cells were then placed in a 37 °C tissue culture incubator at 8.5% CO_2_ for 7–8 days, changing media at approximately 1, 4 and 7 days. Individual cell types were enriched through differential adhesion steps, as described below.

### Enrichment of oligodendrocyte precursor cells

The mixed cultures were placed on an orbital shaker at 220 r.p.m. at 37 °C and 5% CO_2_ overnight to remove the loosely adhered cells (OPCs, microglia and other contaminant cells) from the strongly adhered astrocytes. The media were collected and added to a 100 mm tissue culture dish and placed at 37 °C and 5% CO_2_ for 30 min, to allow preferred adhesion of microglia. The media were collected a second time, now enriched for OPCs, and was centrifuged at 1,200 r.p.m. for 10 min in MGM. After decanting the media, the pellet was resuspended with OPC media^46^, and the total cell number was determined using a hemocytometer. The OPCs were then seeded onto plates under a variety of experimental conditions, as described below.

### Enrichment of astrocytes

After removal of the loosely adherent cells, fresh MGM was added to the T-75 flasks and the cells were placed in an incubator at 37 °C and 5% CO_2_ and used within a few days to prevent the re-population of OPCs and microglia. The astrocytes were removed from the T-75 flasks by digestion with a solution containing 0.4% trypsin, 33 mg ml^−1^ DNaseI and 0.1 mg ml^−1^ EDTA at 37 °C and 5% CO_2_ for 5 min. The reaction was neutralized with excess MGM, and the cells were collected and centrifuged at 1,200 r.p.m. for 10 min. After decanting the media, the pellet was resuspended in MGM and the cells were counted using a hemocytometer. The astrocytes were then seeded under various experimental conditions as described below.

### Primary human astrocytes

The brain tissue from 18- to 20-week-old therapeutically aborted foetuses was used in accordance with ethics approval by the Research Ethics Board at the University of Calgary. The astrocytes were derived and purified by dissociating tissue into single cells, as described elsewhere^48^. The cells were plated in T-75 flasks coated with poly-L-ornithine (10 μg ml^−1^), in minimal essential medium supplemented with 10% fetal bovine serum, 1 mM sodium pyruvate, 0.1% dextrose and 1% penicillin/streptomycin. The cultures were passaged seven to eight times over 4–5 weeks, depleting neurons and achieving an astrocyte purity of 95%.

### Growth of OPCs on a purified CSPG mixture

To determine the effects of CSPGs on OPC growth *in vitro*, flat-bottom 96-well plates were first coated with 10 μg ml^−1^ poly-L-lysine overnight, followed by a rinsing with water. Purified CSPGs (Millipore CC-117), a commercial mixture containing neurocan, phosphacan, versican and aggrecan, were coated onto the 96-well plates at a concentration of 10 ng ml^−1^–10 μg ml^−1^ diluted in sterile water for 3–4 h, followed by a rinsing with water. The control wells contained poly-L-lysine alone or bovine serum albumin (1–10 μg ml^−1^). Enriched OPCs were seeded at a density of 5.0–10.0 × 10^4^ cells per well in OPC media (*n*=4 wells per condition), and the plates were incubated at 37 °C and 8.5% CO_2_ for 18–24 h for most experiments. The cells were fixed with 4% ice-cold paraformaldehyde at 4 °C for 10 min, rinsed with phosphate-buffered saline (PBS), and stored at 4 °C until processed for immunocytochemistry. In other experiments, OPCs were seeded onto astrocyte-secreted ECM.

### Immunocytochemistry

All the steps were conducted at room temperature. For intracellular proteins, such as GFAP in astrocytes, the cells were permeabilized using 0.25% Triton X-100 in PBS for 10 min. Probing for membrane components, such as Iba1 on microglia and sulfatide O4 on oligodendrocyte lineage cells, did not require permeabilization. Non-specific antibody interactions were blocked using 20% normal goat serum in PBS for 60 min. Primary antibodies were diluted in normal goat serum and incubated for 60 min. The cells were washed with PBS and secondary antibodies diluted in normal goat serum, alongside Nuclear yellow (1:1,000 dilution) for detecting cell nuclei, were added for 60 min. The cells were washed with PBS and stored at 4 °C in PBS until ready for imaging. Primary antibodies used for immunocytochemistry are listed in Supplementary Table 1.

### ImageXpress acquisition and MetaXpress analysis

Plates containing cells processed for immunocytochemistry were imaged with a Molecular Devices ImageXpress high-content imaging system. The images were collected at × 10 magnification from 12 sites per well, with appropriate absorption and emission wavelengths for each secondary antibody. Once acquired, images were processed through MetaXpress analysis software. The first software module used was ‘multiwavelength cell scoring', which uses fluorescence intensity and *x*-*y* coordinates to measure co-localization of markers from different channels within individual cells. The second software module used was ‘neurite outgrowth', which uses fluorescence intensity and user-entered estimate cell and process sizes to quantify the number of cells per field, as well as a measure of process outgrowth per cell. The data from 12 images were averaged to a single data point for each replicate well^36^.

### Live–dead cell assay

Astrocyte viability in the presence of various pharmacological agents was determined using a live–dead cell assay. Cell-permeant Nuclear blue (1:10 dilution) and live cell-impermeant propidium iodide (10 μg ml^−1^) were added to cells growing in MGM for 30 min. The cells were then imaged on the ImageXpress machine and images analysed using MetaXpress multiwavelength cell scorer.

### Astrocyte-secreted extracellular matrix

To generate an anchored astrocyte ECM^36^, the astrocytes were seeded at a density of 1.0 × 10^5^ cells per well on flat-bottom 96-well plates that were previously coated overnight with 10 μg ml^−1^ poly-L-lysine, followed by a rinse with water. The cells were incubated at 37 °C and 5% CO_2_ for 7 days in MGM, changing media every other day. The cells were then rinsed with PBS and treated with versene for 30 min at 37 °C and 5% CO_2_, using a 200 μl micropipettor to dislodge the cells. The cellular debris was removed with a PBS rinse, and confirmed under a phase-contrast microscope. The deposited ECM anchored to the 96-well plate was kept hydrated with PBS at 4 °C until ready to be seeded with OPCs.

In some experiments, astrocyte ECM was collected in the media^34^. This was accomplished by seeding astrocytes at 1.0 × 10^6^ cells per well of six-well plates that were not poly-L-lysine coated, to minimize secreted ECM adherence to the plate bottom. The cells were kept for 2 days without media changes. The media were then pooled from three triplicate wells (∼5.5 ml total) and centrifuged at 5.0 × 10^3^ × g in a 100 kDa cutoff molecular weight centrifugal filter, concentrated 10–20 × and stored at −80 °C in the presence of a protease inhibitor cocktail.

### Western blot

For lysates of mouse spinal cord, the animals were first killed with carbon dioxide asphyxiation, and the spinal cords were rapidly removed and snap frozen in liquid nitrogen. A 2 mm piece of tissue containing the site of lysolecithin injection was lysed in 10 times volume of 40 mM Tris-HCL, pH 7.6, containing 40 mM sodium acetate and a protease inhibitor cocktail with a 5 s pulse of an ultrasonicator^49^. Disrupted tissue was centrifuged at 14.0 × 10^3^ × *g* for 10 min, and the supernatant was transferred to a new tube. The total protein was quantified using a Bradford assay.

For western blotting, some samples were first digested with 0.2 U ml^−1^ chondroitinase ABC for 3–24 h at 37 °C to remove chondroitin sulfate sidechains. The samples were heat denatured with SDS at 95 °C for 5 min, and loaded into 3–8% tris-acetate pre-cast gels. The gels were electrophoresed at 150 V for 60 min and transferred to a 0.2 μm polyvinylidene fluoride membrane at 250 mA for 60 min. The membranes were blocked with 10% skim milk in 0.5 tween in tris-buffered saline (TBS-T), and primary antibodies (Supplementary Table 1) incubated overnight at 4 °C. The membranes were washed 5 × 5 min with TBS-T and HRP-conjugated secondary antibodies were incubated for 120 min. The membranes were washed again for 5 × 5 min with TBS-T and subject to enhanced chemiluminescence and developed. Images were cropped for presentation in Figs 3, 5, Supplementary Figs 4 and 5, and full sized versions are presented in Supplementary Figs 8–11.

### Proliferation of splenocytes

The spleens were isolated from 8-10-week-old female C57BL/6 mice and homogenized. The cells were isolated by Ficoll gradient and spun for 30 min at 1,800 r.p.m. The cell suspension was removed and the cells were washed once with PBS. The cell pellet was subsequently re-suspended in Roswell Park Memorial Institute media containing 10% fetal bovine serum, 1% penicillin/streptomycin, 1% sodium pyruvate and 1% L-glutamine. The live cells were counted with Trypan blue, and the cells were plated at 2.5 × 10^5^ cells per well in a round-bottom 96-well plate and activated with 1,000 ng ml^−1^ plate-bound anti-CD3 and 1,000 ng ml^−1^ anti-CD28 suspended in media to preferentially activate T lymphocytes. Fluorosamine was added at final concentrations of 1, 10 and 100 μM. The cells were kept at 37 °C and 5% CO_2_ for 30 h, after which 10 μl of ^3^H-thymidine at a concentration of 1 μCi per well was added for 18 h. The media containing thymidine were harvested onto filter mats using a cell harvester and mats were allowed to dry for 24 h. The results were read by liquid scintillation counts.

### Lysolecithin demyelination

All *in vivo* experiments were performed with 8–12-week-old female C57BL/6 mice in accordance with ethical animal care guidelines by the Animal Care Committee at the University of Calgary. Three to six animals per group were chosen for experiments based on past experience with group variability. Experimental demyelination was produced using the toxin lysolecithin as previously described^37^. The animals were anaesthetized with an intraperitoneal injection of ketamine (200 mg kg^−1^) and xylazine (10 mg kg^−1^). The back was shaved with a razor, and hair clippings removed with 70% ethanol. The surgical field was wiped with iodine, and buprenorphine (0.05 mg kg^−1^) was administered subcutaneously as an analgesic. After positioning the animal in a stereotactic frame, a midline incision was performed, followed by separation of the underlying muscle and adipose tissue with retractors. The bony outgrowth of the thoracic-level(T)2 vertebrae was identified as a landmark, and the connective tissue between T3 and T4 was removed with spring scissors, revealing the spinal cord. The thick layer of dura was then cleared using lateral scrapes with a 30-gauge metal needle. The grey–white matter boundaries on either side of the dorsal columns were used to visualize the approximate midline of the cord.

The spinal cords were pierced at either side of the midline with either a 34 gauge 12° bevelled needle, or by a pulled glass capillary, attached to a 10 μl syringe filled with 1% lysolecithin in PBS. The needles were lowered 1.3 mm from the dorsal surface of the spinal cord at an angle of 5°, to be positioned in the ventrolateral white matter. Lysolecithin was then injected at a rate of 0.25 μl per minute for 2 min, for a total volume of 0.50 μl. The needles were held in place for 2 min to prevent the backflow of solution. The needles were then retracted, and the incision sites were sutured closed. The animals were kept in heated recovery chambers until they awoke from anaesthesia, and returned to their cages.

Sham surgeries were performed identically as described, with the injection of PBS instead of lysolecithin into the ventral white matter.

### Histology

To study different aspects of the de- and remyelination response, the animals were killed at 7 or 14 days after injection of lysolecithin. The animals were transcardially perfused with 20 ml room temperature PBS followed by 20 ml ice-cold 4% paraformaldehyde in PBS. The spinal cords were isolated and post-fixed in 4% paraformaldehyde overnight at 4 °C, cryoprotected with 30% sucrose at 4 °C for 72 h, cut into 3 mm pieces with the injection site in the centre and frozen in OCT compound in moulds (up to five cords per mould) resting on a mixture of dry ice and 2-methyl-butane. The blocks were stored at −80 °C until ready to section.

The cords were cut into 20 μm coronal sections over a series of 10 sets of slides, such that adjacent sections on a single slide were 200 μm apart. The slides were kept at −20 °C until ready to process for histology.

### Eriochrome cyanine

The first series of tissue was exposed to eriochrome cyanine, which stains myelin lipids with a blue colour. All the steps were performed at room temperature. The slides were air-dried for 30 min, then placed in clearing agent for 1 min followed by rehydration in graded ethanol solutions (100%, 95%, 90%, 70%, 50%, water) for 1 min each. The slides were placed in eriochrome cyanine solution for 15 min, washed with water for 1 min, differentiated with 0.5% ammonium hydroxide for 10 s and washed with water again for 1 min. The sections were dehydrated in graded ethanol solutions (water, 50%, 70%, 90%, 95%, 100%), placed in clearing agent for 1 min and coverslipped with mounting media. The images were taken on an Olympus bright-field microscope to visualize the size and location of lesions to guide image acquisition with other series of tissue used for immunohistochemistry.

### Immunohistochemistry

Room-temperature-thawed slides were hydrated with PBS. The tissue was permeabilized with 0.25% Triton X-100 in PBS for 30 min and rinsed with PBS. In the instances of CSPG core protein staining, chondroitinase ABC (0.2 U ml^−1^ in PBS) was added for 30 min to remove chondroitin sulfate sidechains, followed by a PBS rinse. The sections were post-fixed in methanol at −20 °C for 10 min, rinsed with PBS and blocked with 10% horse serum containing 1% bovine serum albumin and 0.1% cold fish-skin gelatin. Primary antibodies (Supplementary Table 1) were incubated overnight, followed by a PBS rinse; and secondary antibodies alongside Nuclear yellow (1:1,000) were incubated for 60 min, followed by a final rinse with PBS and mounting with gelvatol. Images (× 10 and × 60) were taken on an Olympus FluoView FV10i confocal microscope.

### Semithin sectioning

The animals were killed as described above, with a perfusion of 2.5% glutaraldehyde and 4% paraformaldehyde in PBS. The spinal cords were removed and post-fixed overnight at 4 °C in the same fixative. The cords were rinsed three times with 0.1 M cacodylate, pH 7.2 and then fixed with 1% osmium tetroxide in 0.1 M cacodylate for 60 min at room temperature. The cords were dehydrated in graded ethanol and embedded in Epon resin. The blocks were cut on a Leica EM UC6 ultramicrotome, sampling cords every 500 μm. The sections were stained briefly with 1% toluidine blue and 2% borax. The sections were mounted with acrytol and imaged at × 40 and × 100 with an Olympus bright-field microscope. The relative amount of remyelination present was ranked by a blinded examiner. ImageJ was subsequently used to quantify the area of lesion occupied by myelinated axons by a different blinded examiner; total epicentre lesion area was also quantified.

### Experimental autoimmune encephalomyelitis

Eight- to twelve-week-old female C57BL/6 mice were anaesthetized with an intraperitoneal injection of ketamine (200 mg kg^−1^) and xylazine (10 mg kg^−1^). Myelin oligodendrocyte glycoprotein (MOG) peptide 35–55 was obtained from the University of Calgary peptide facility. An amount of 50 μg MOG per animal was emulsified in 100 μl Complete Freund's Adjuvant (CFA) supplemented with *Mycobacterium tuberculosis.* The mice received one 50 μl injection per hind flank for a total of 50 μg MOG administration in 100 μl of emulsion. The mice were then injected intraperitoneally with 300 ng pertussis toxin (PTx) on the day of immunization (day 0) and day 2. All the animals were weighed and scored daily using a previously described 15-point scale^50^. In brief, the degree of disability of each mouse's tail, fore- and hind-limbs was individually assessed and summed for that animal. For tail function, 0 refers to no physical signs, 1 is partial paralysis and 2 is complete paralysis. For each limb, 0 refers to no symptoms, 1 to a weak and abnormal walk, 2 to a dragging limb that still has movements and a score of 3 is full paralysis of that limb. Following this scheme, a fully paralysed quadriplegic animal (with a score of 2 in tail function, and 3 in each limb) would be given a score of 14. A death attributable to EAE would be given a score of 15.

### Quantitative real-time polymerase chain reaction

Fresh cervical spinal cord segments were extracted from EAE mice on day 26, flash frozen in liquid nitrogen, and stored in −80 °C. All procedures were performed with RNAse free material and solutions. Tissue was lysed in 1 ml of TRIzol, using 22 G and 25 G needles to shear DNA and fine-mince the sample. RNA was precipitated with RNeasy Mini Kit columns. A spectrophotometer assessed RNA for concentration and quality (A260/A280 ratio). The samples were treated with DNAse according to the manufacturer's (Promega) instructions. RNA was reverse transcribed to cDNA using Superscript II Reverse Transcriptase (Invitrogen). All the primers used in this study were purchased from Qiagen: Aggrecan (QT00175364), Neurocan (QT00158823), Versican V0/V1 (QT01889475) with GAPDH as a housekeeping control (QT01658692). The transcripts were quantified by quantitative real-time PCR on an iCycler using RT2 Real Time SYBR Green/Fluorescein PCR Master Mix. The relative expression levels between genes were calculated using a comparative cycle threshold method, with expression levels normalized to GAPDH.

### Statistics

All statistics were performed using Prism 6.0 software. Data sets were analysed with Student's *t*-test, Mann–Whitney *U*-test or analysis of variance with multiple comparisons between groups with an alpha of 0.05.

## Additional information

**How to cite this article:** Keough, M. B. *et al*. An inhibitor of chondroitin sulfate proteoglycan synthesis promotes central nervous system remyelination. *Nat. Commun.* 7:11312 doi: 10.1038/ncomms11312 (2016).

## Supplementary Material

Supplementary Information Supplementary Figures 1-11, Supplementary Tables 1-3 and Supplementary Methods.
