# Supplementary material for: An inhibitor of chondroitin sulfate proteoglycan synthesis promotes central nervous system remyelination
Source: Nat Commun. 2016 Apr 26;7:11312. doi: 10.1038/ncomms11312 (PMC4853428; doi:10.1038/ncomms11312)
Supplement: Supplementary Information — Supplementary Figures 1-11, Supplementary Tables 1-3 and Supplementary Methods. [file ncomms11312-s1.pdf]

Supplementary Figure 1 Purity of enriched oligodendrocyte precursor cells. (**a**) Representative immunohistochemistry images of enriched OPCs plated for 18-24h and stained for O4 and the microglial marker Iba1. (**b**) Quantification of cell numbers reveal that approximately 75% of cells are O4+, and approximately 20% are Iba1+. Approximately 5% of nuclear yellow positive nuclei are neither O4 nor Iba1+ cells, and are likely other contaminating cells, or oligodendrocyte lineage cells that are not expressing O4. Results are presented as 4 replicate wells of an individual experiment that was conducted once. Error bars are mean ± s.d. Scale bar = 25 μm.

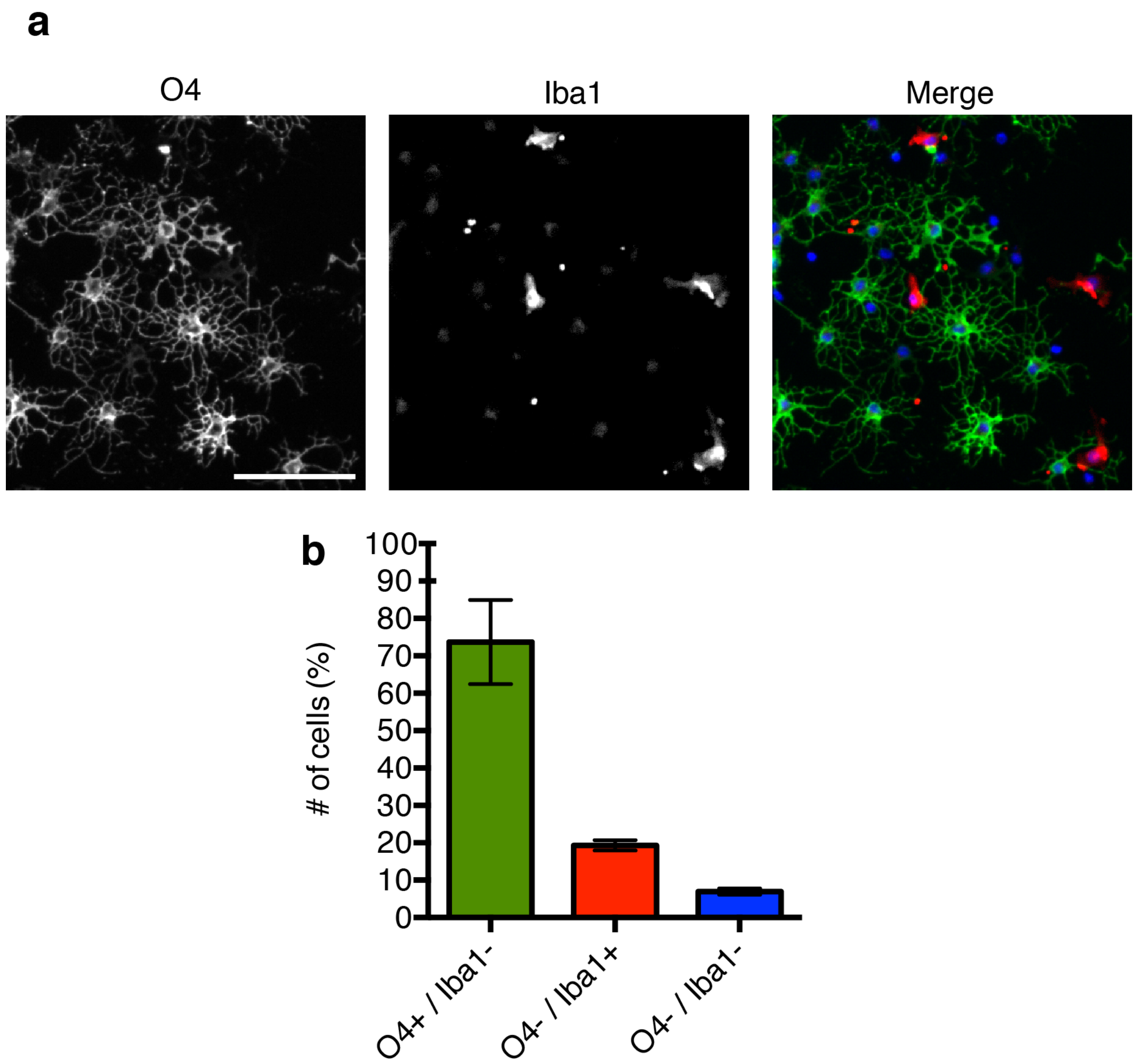

Supplementary Figure 2 Synthesis of chondroitin sulfate and proposed mechanism of fluorosamine. CSPG side chains are synthesized from hydroxyl containing amino acids on the protein core (right) first with a linker region of xylose followed by two galactose sugars (blue). Repeating units of glucuronic acid (GlcA) and N-acetylgalactosamine (GalNAc) make up the remainder of the chain. GalNAc is converted from N-acetylglucosamine (GlcNAc) by the enzyme 4-epimerase. Fluorosamine (depicted in red) is hypothesized to inhibit the conversion of GlcNAc to GalNAc, reducing available metabolites for side chain synthesis.

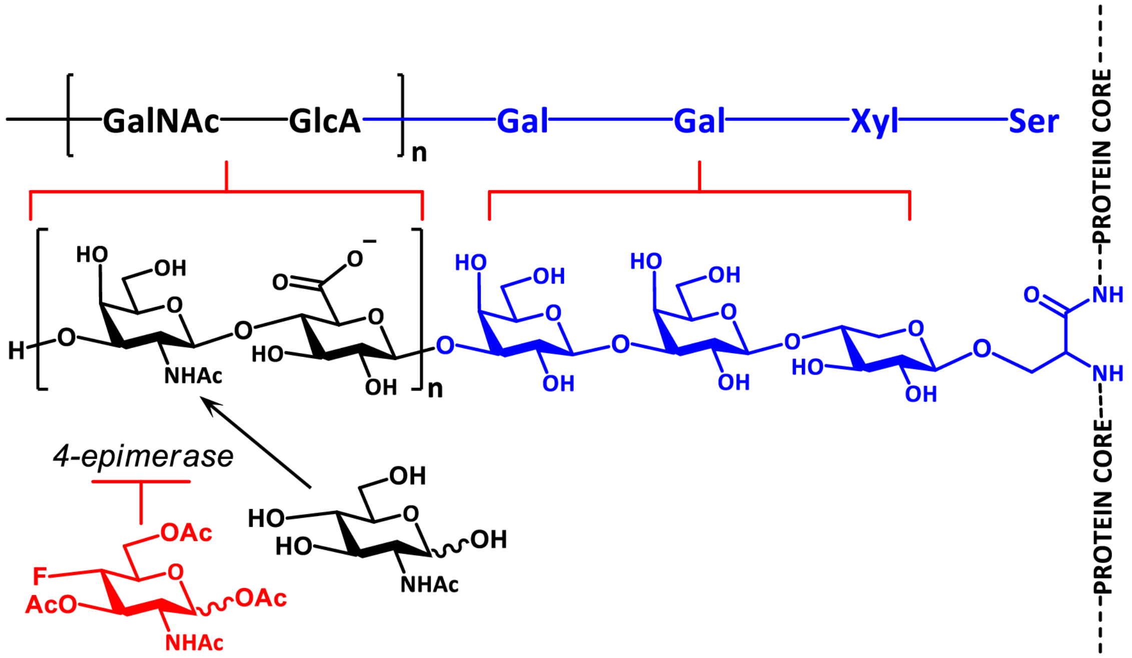

Supplementary Figure 3 Chemical synthesis reaction strategy for 4-F-GlcNAc (3), fluorosamine (4), and the 1-hydroxy (5) propanoate ester (6) compounds.

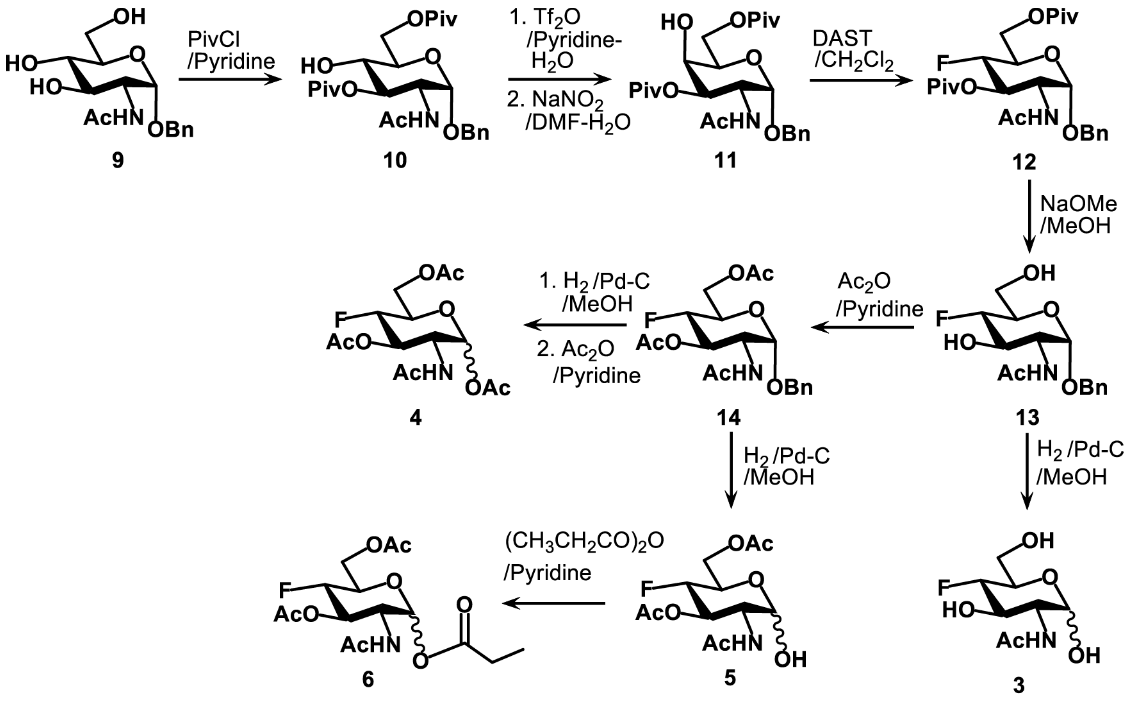

Supplementary Figure 4 Fluorosamine reduces CSPGs in human astrocytes and is non-toxic in culture. (**a**) Western blot of CSPG core protein (MAB2030) from human astrocyte conditioned media shows an increase in signal intensity with TGF-B1 stimulation. Treatment with fluorosamine reduces this effect to a greater degree than the non-acetylated 4-F-GlcNAc. (**b**) A propidium iodide live/dead cell uptake assay from murine astrocytes shows that fluorosamine does not significantly increase the amount of dead cells 48h after treatment, with peroxide treatment as a positive control for cell death. Results are represented as 3 replicate wells of an individual experiment (**a**) or 4 replicate wells of an individual experiment (**b**) that was conducted twice (**a**) or three times (**b**). *** P < 0.001, one-way ANOVA of % dead cells with Dunnett's post-hoc test (respective of control). Error bars are mean ± s.e.m.

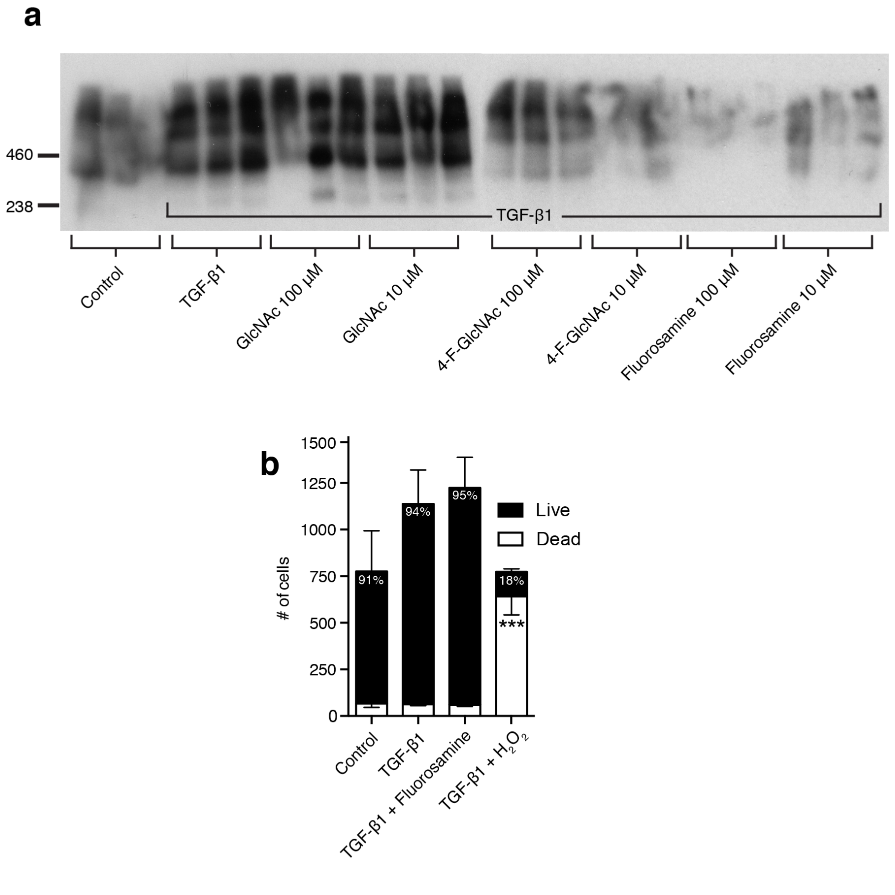

Supplementary Figure 5 Fluorosamine is likely an alternative substrate for N-acetylglucosamine. A western blot of CSPG core proteins (MAB2030) from murine astrocyte media 48h after treatment with different ratiometric concentrations of acetylated glucosamine (Ac-GlcNAc) and fluorosamine shows that a ratio of at least 10:1 of fluorosamine relative to Ac-GlcNAc is required to observe a reduction in signal intensity. Lanes are pooled from 3 replicate wells of an individual experiment.

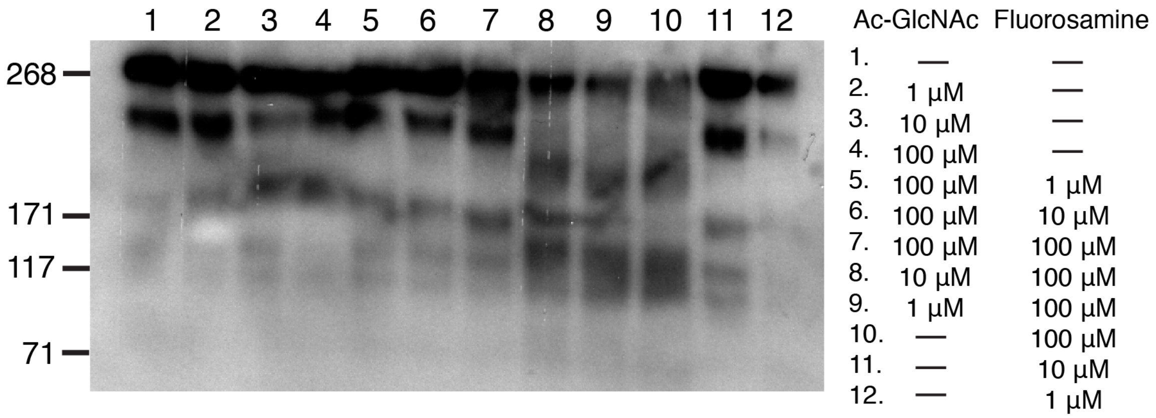

Supplementary Figure 6 Chemical synthesis reaction strategy for 4-methoxy (7) and 1-hydroxy-4-chloro (21) derivatives.

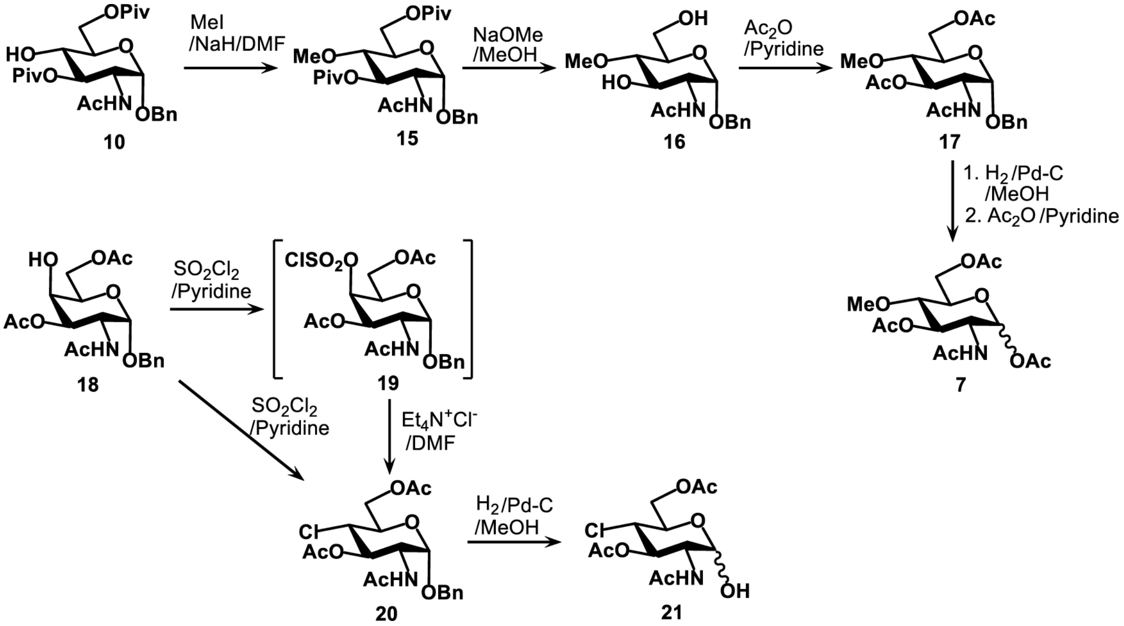

Supplementary Figure 7 Detection of remyelinated axons. 100X magnification toluidine blue stained semithin section of lysolecithin demyelinated spinal cord. Remyelinated axons were defined as those circumscribed by thin sheaths that were much fainter in intensity (colored in pink, right) compared to those that were circumscribed by a thick dark sheath, presumably unaffected by lysolecithin injection. Fraction of remyelinated area is calculated by dividing the lesion area occupied by remyelinated axons (area of pink color) by the total lesion area. Scale bar = 10 μm.

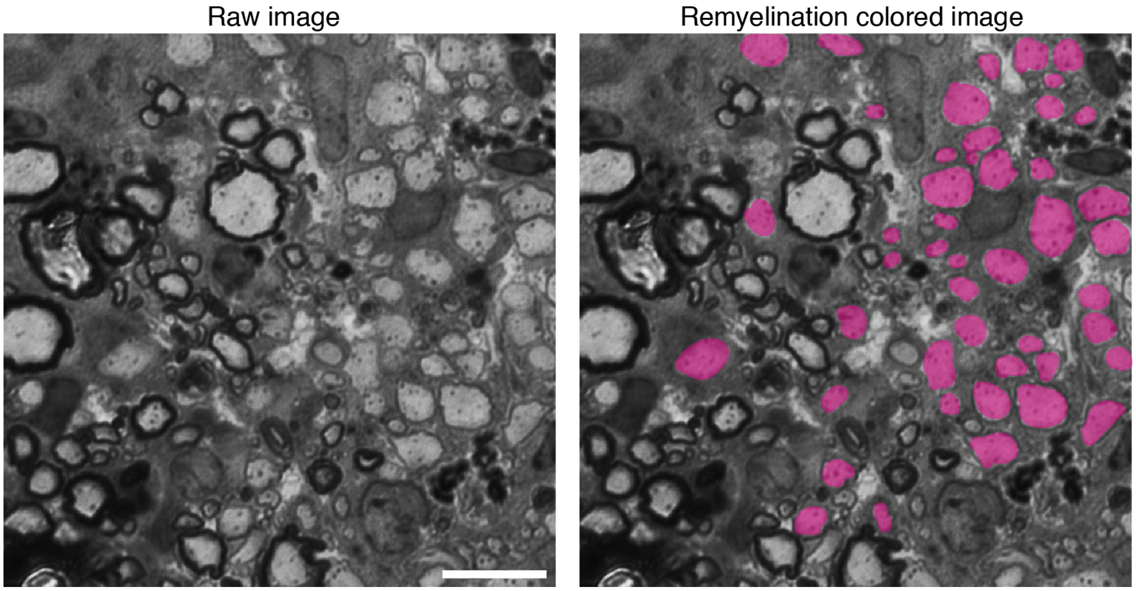

Supplementary Figure 8 Full size western blots corresponding to Fig. 3 with molecular weight ladder values added.

a

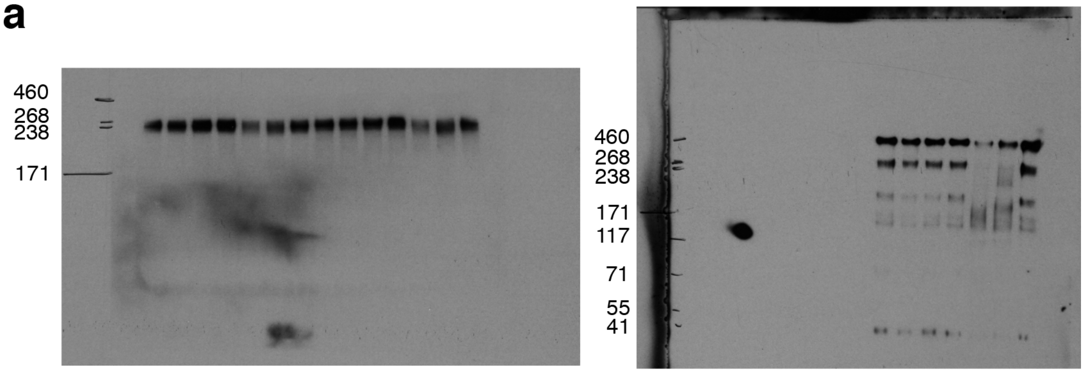

b

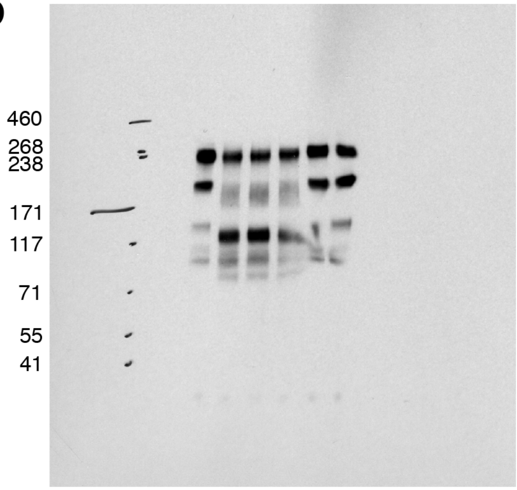

Supplementary Figure 9 Full size western blots corresponding to Fig. 5 with molecular weight ladder values added.

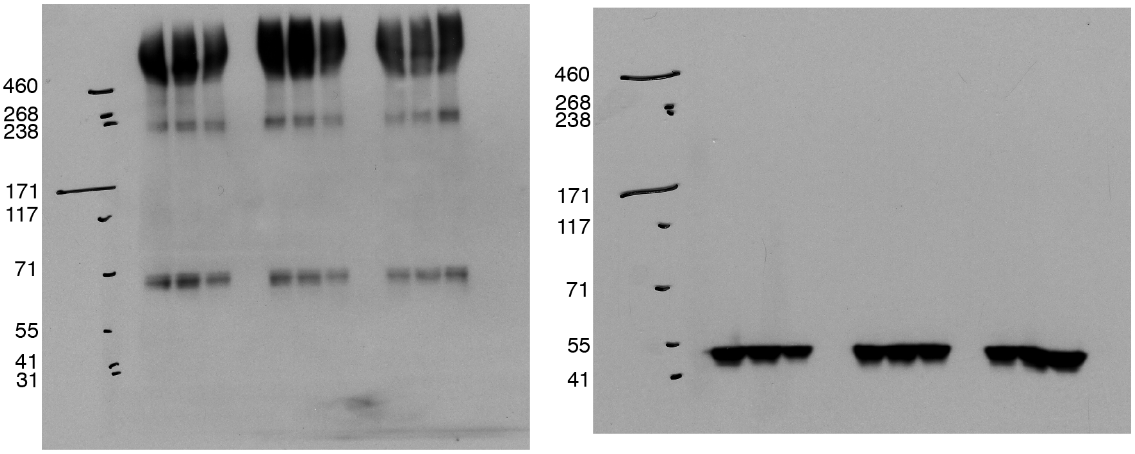

Supplementary Figure 10 Full size western blot corresponding to Supplementary Fig. 4 with molecular weight ladder values added

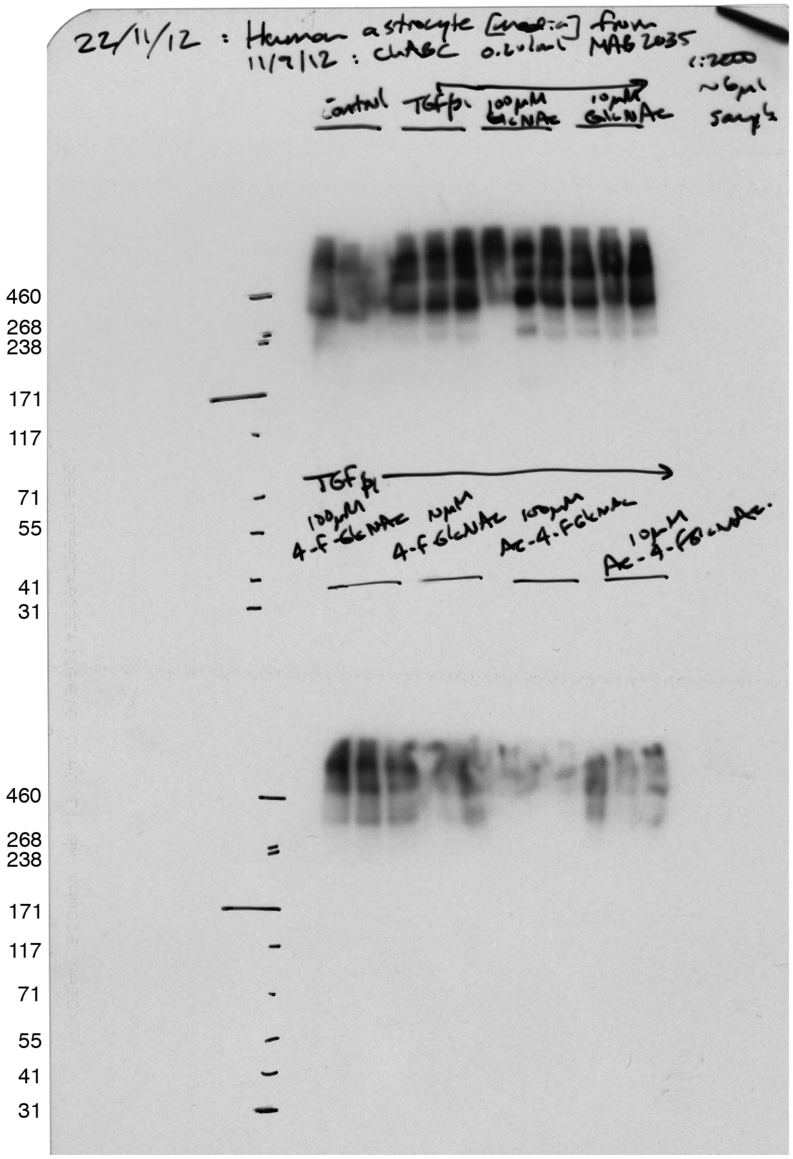

Supplementary Figure 11 Full size western blot corresponding to Supplementary Fig. 5 with molecular weight ladder values added

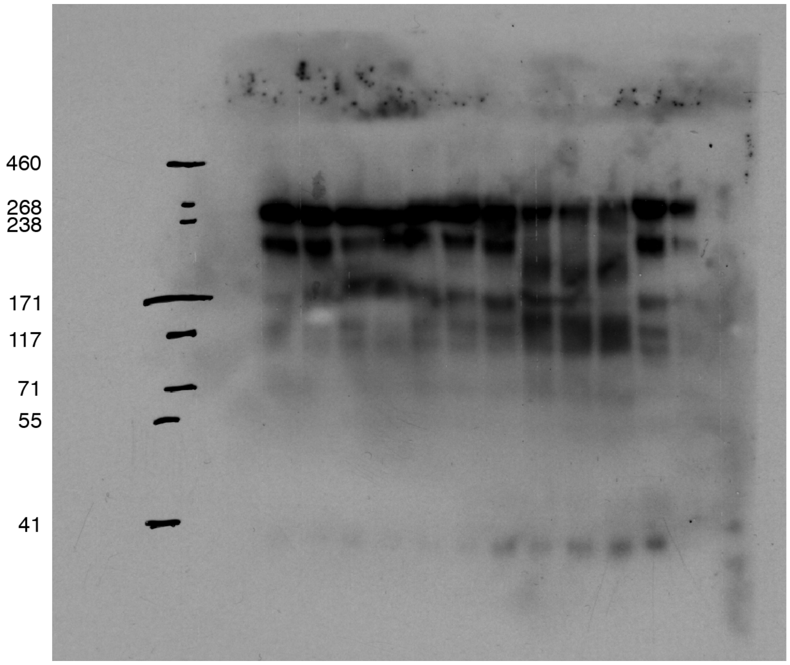

Supplementary Table 1 Product information and protocol specifics for antibodies used.

Antibody	Company	Catalog	Species	Dilution	Use
Stub chondroitin sulfate	EMD Millipore	MAB2030	Mouse	1:2000	W.B.
chondroitin sulfate	Cosmobio	CAC-NU-07-001	Mouse	1:2000	W.B.
Versican GAG α	EMD Millipore	AB1032	Rabbit	1:100	I.H.C.
Versican GAG β	EMD Millipore	AB1033	Rabbit	1:100	I.H.C.
Aggrecan	EMD Millpore	AB1031	Rabbit	1:100	I.H.C.
GFAP	Sigma	G3893	Mouse	1:1000	I.H.C.
PDGFR α	R&D Systems	AF1062	Goat	1:100	I.H.C.
CC-1	EMD Millipore	OP80	Mouse	1:200	I.H.C
Olig2	EMD Millipore	AB9610	Rabbit	1:200	I.H.C.
O4	EMD Millipore	MAB345	Mouse	1:250	I.C.C.
Iba1	Wako	019-19741	Rabbit	1:250	I.C.C.

Supplementary Table 2 Examples of drug classes contained in the screen.

**Drug Class**
analgesic
anthelmintic
antiandrogenic
antianemic
antianginal
antiarrhythmic
antiasthmatic
antibacterial
anticholinergic
anticonvulsant
antidepressant
antidiabetic
antidyskinetic
antiemetic
antifungal
antihistaminic
antihyperlipidemia
antihyperglycemia
antihypertensive
anti-inflammatory
antimalarial
antineoplastic
antipsychotic
antiulcerative
antiviral
bronchodilator
diuretic
immunosuppressant
muscle relaxant
vasodilator

Supplementary Table 3 Quantification of OPC process outgrowth on CSPGs in the presence of 245 orally available Health Canada approved medicines. As noted in Methods, the extent of process outgrowth of cells in 96-well plate was acquired by ImageXpress® and quantitated by MetaXpress® software. Results are presented as averages (% change relative to CSPG) and standard deviations from 4 wells of an individual drug treatment. Several of the compounds were toxic (large negative values that were statistically significant) and highlighted in red. All compounds were used at 10 μM concentration.

Drug	Mean (% relative to CSPG)	St.Dev	significance
Acebutolol Hydrochloride	5.38	6.50	
Acetaminophen	-4.36	2.50	
Acetazolamide	-12.99	7.16	
Acetylcysteine	-1.05	4.50	
Acyclovir	12.75	10.47	
Allopurinol	2.88	3.50	
Almotriptan	-2.26	4.12	
Amantadine Hydrochloride	-4.38	3.79	
Amikacin sulfate	-5.67	8.17	
Amiloride hydrochloride	5.83	4.02	
Amiodarone Hydrochloride	-2.13	4.00	
Amitriptyline Hydrochloride	6.60	3.65	
Amlodipine Besylate	-89.14	2.10	****
Amoxicillin	-22.50	18.71	
Amphotericin B	-40.48	8.58	****
Antipyrine	-4.06	2.65	
Aspirin	-13.75	5.21	**
Atenolol	-9.44	4.54	
Atorvastatin Calcium	-15.01	7.32	*
Atovaquone	7.13	7.06	
Azathioprine	-10.66	6.28	
Azithromycin	-30.13	5.08	
Baclofen	-10.80	8.18	
Benazepril Hydrochloride	-1.90	8.81	

Benserazide hydrochloride	-24.73	5.75	
Bethanechol chloride	-15.65	7.68	
Bezafibrate	-4.55	3.95	
Bisacodyl	-3.85	5.15	
Brompheniramine Maleate	-5.48	13.12	
Budesonide	-13.93	12.64	
Bumetanide	2.22	9.88	
Bupropion	-0.14	5.99	
Busulfan	-1.18	4.77	
Candesartan Cilextil	-6.69	6.82	
Captopril	-8.91	3.52	
Carbachol	-10.17	6.99	
Carbamazepine	-15.78	9.76	
Carvedilol Tartrate	0.23	6.44	
Cefaclor	-3.79	3.34	
Cefadroxil	0.96	4.26	
Cephalexin	-5.00	8.10	
Chlopromazine	-72.30	1.56	****
Chlorpheniramine (S) maleate	-12.33	3.28	
Chlorpropamide	-1.17	3.66	
Chlorthalidone	-7.04	6.80	
Ciclosporine	-18.56	9.25	
Cimetidine	-16.48	4.11	****
Ciprofloxacin	1.68	7.22	
Clarithromycin	-3.06	6.28	
Clindamycin Hydrochloride	-0.31	3.19	
Clomipramine Hydrochloride	-19.95	2.87	***
Clonidine hydrochloride	-7.21	9.40	
Clopidogrel Sulfate	4.39	4.71	
Clotrimazole	-10.73	6.09	*
Cloxacillin sodium	-10.65	5.33	
Clozapine	-11.49	8.96	
Colchicine	-82.09	4.13	****
Cresol	-14.69	9.65	*
Cromolyn Sodium	-18.90	5.16	**
Cyclobenzaprine hydrochloride	18.50	11.56	
Cyclophosphamide Hydrate	-7.38	6.14	
Danazol	-11.07	29.32	
Dapsone	2.66	2.62	
Dequalinium Chloride	-77.71	5.54	****

Desipramine hydrochloride	-3.31	11.01	
Dextromethorphan hydrobromide	-13.13	3.69	
Diazoxide	-11.76	9.83	*
Diclofenac sodium	-7.81	7.22	
Diflunisal	-5.25	4.36	
Digoxin	-31.36	5.11	
Diltiazem Hydrochloride	-5.00	6.83	
Dimenhydrinate	-7.58	8.69	
Diphenydramine hydrochloride	-20.38	16.57	
Diphenylpyraline hydrochloride	-19.02	6.11	
Dipyridamole	-15.76	15.00	
Disopyramide Phosphate	4.97	9.38	
Doxepin Hydrochloride	-3.32	5.93	
Doxycycline Hydrochloride	12.03	11.01	
Doxylamine Succinate	0.63	2.05	
Edrophonium Chloride	0.03	8.96	
Enalapril Maleate	0.37	10.68	
Ergonovine Maleate	2.43	5.16	
Erythromycin Estolate	-16.78	1.65	**
Ethambutol hydrochloride	-9.78	10.79	
Ethosuximide	-10.22	3.69	
Etodolac	1.00	6.10	
Ezetimibe	2.91	2.46	
Famciclovir	4.36	6.21	
Famotidine	-3.56	3.79	
Fenofibrate	-4.64	2.89	
Flunarizine Hydrochloride	14.61	6.60	
Fluoxetine	-0.70	3.86	
Fluphenazine Hydrochloride	-91.17	3.67	****
Flurbiprofen	-5.71	4.11	
Fosfomycin	-1.82	8.43	
Furosemide	7.47	5.33	
Galantamine Hydrobromide	-6.03	5.70	
Gemfibrozil	-9.55	9.43	
Gliclazide	-1.13	5.04	
Glyburide	-9.92	11.45	
Guaifenesin	5.73	3.64	

Haloperidol	-19.30	7.04	**
Hexylresorcinol	-9.06	13.15	
Hydralazine Hydrochloride	4.23	3.48	
Hydrochlorothiazide	-11.10	2.41	
Hydroxychloroquine Sulfate	-8.38	7.24	
Hydroxyurea	-0.90	5.83	
Hydroxyzine Pamoate	3.15	10.37	
Ibuprofen	3.90	2.78	
Imipramine Hydrochloride	12.24	1.60	
Indapamide	0.71	7.68	
Indomethacin	-6.48	8.63	
Ipratropium Bromide	-5.37	3.50	
Irbesartan	-6.44	5.76	
Isoniazid	3.50	8.18	
Isosorbide Dinitrate	-3.61	5.19	
Ketoconazole	1.55	5.27	
Ketoprofen	-5.55	3.47	
Ketorolac Tromethamine	-5.56	6.97	
Ketotifen Fumarate	-68.34	3.03	****
Labetalol Hydrochloride	3.57	11.32	
Lactulose	-5.73	3.69	
Lansoprazole	10.77	5.39	
Leucovorin Calcium	-5.84	1.89	
Levodopa	-49.26	2.56	****
Levofloxacin	3.47	4.38	
Liothyronine Sodium	-0.11	8.72	
Lisinopril	-2.74	7.83	
Loperamide Hydrochloride	-4.55	2.61	
Loratadine	5.03	5.07	
Losartan	-5.44	3.42	
Lovastatin	3.61	4.60	
Loxapine Succinate	-9.06	5.09	
Maprotiline Hydrochloride	-82.48	2.51	****
Mebendazole	-37.11	3.51	****
Mefenamic Acid	-8.49	6.81	
Mefloquine	-56.40	10.00	****
Meloxicam	12.67	10.08	
Memantine Hydrochloride	-1.62	6.22	
Mercaptopurine	3.96	2.38	
Methazolamide	9.39	5.74	

Methenamine	-2.45	2.60	
Methocarbamol	5.02	4.78	
Methotrexate	-1.76	7.85	
Methoxsalen	1.21	6.68	
Methyldopa	-3.11	7.12	
Metoclopramide Hydrochloride	-4.63	3.84	
Metolazone	-8.41	2.49	
Metoprolol Tartrate	-8.16	4.30	
Metronidazole	-3.18	5.14	
Midodrine Hydrochloride	-4.79	2.75	
Minoxidil	1.08	6.66	
Mitoxanthrone Hydrochloride	-82.62	2.33	****
Modafinil	5.10	5.86	
Moxifloxacin Hydrochloride	-9.24	7.96	
Mycophenolic Acid	-66.23	8.63	****
Nabumetone	-7.21	4.96	
Nadolol	-6.86	2.60	
Naloxone Hydrochloride	0.58	2.89	
Naltrexone Hydrochloride	-4.58	11.14	
Naproxen(+)	-2.02	5.69	
Neostigmine Bromide	4.30	3.67	
Nifedipine	7.22	12.31	
Nilutamide	-8.87	3.59	
Nimodipine	7.34	4.46	
Nitrofurantoin	-13.05	4.98	
Norfloxacin	10.55	5.42	
Nortriptyline	-68.69	10.17	****
Nylidrin Hydrochloride	-7.25	6.35	
Olmesartan Medoxomil	-6.69	5.28	
Orlistat	1.61	8.58	
Orphenadrine Citrate	16.50	6.93	
Oxcarbazepine	-12.69	5.16	*
Paromomycin Sulfate	-7.12	4.76	
Pentoxifylline	-8.13	3.46	
Periciazine	14.85	15.08	
Perindopril Erbumine	-8.74	2.08	
Perphenazine	0.67	6.76	
Phenazopyridine Hydrochloride	1.36	3.74	

Phenelzine Sulfate	-0.98	6.73	
Phenytoin Sodium	-1.64	4.52	
Pimozide	-19.25	14.07	
Pindolol	-9.13	4.14	
Pioglitazone Hydrochloride	-7.60	4.35	
Piroxicam	-8.52	3.49	
Potassium p-aminobenzoate	-9.67	5.22	
Pravastatin Sodium	-15.21	6.70	*
Pregabalin	6.40	8.30	
Primaquine diphosphate	5.62	5.21	
Primidone	-8.01	1.32	
Probenecid	-11.40	2.17	*
Procainamide Hydrochloride	-9.19	3.08	
Prochlorperazine Edisylate	-83.99	4.46	****
Procyclidine Hydrochloride	10.00	7.83	
Promethazine Hydrochloride	-18.28	5.31	**
Propafenone Hydrochloride	17.83	13.12	
Propranolol Hydrochloride (+/-)	2.46	8.27	
Propylthiouracil	1.80	7.26	
Pseudoephedrine hydrochloride	-10.50	5.26	
Pyrantel pamoate	-11.25	5.83	
Pyrazinamide	11.63	3.68	
Pyridostigmine Bromide	-2.19	2.51	
Quinapril Hydrochloride	1.00	8.23	
Quinine Sulfate	-12.34	9.24	
Ranitidine	3.23	6.82	
Rifampin	5.54	6.65	
Rifaximin	-0.71	6.30	
Riluzole	-2.86	7.46	
Rosiglitazone	-18.43	6.19	***
Rosuvastatin	-7.09	10.32	
Sertraline Hydrochloride	-82.51	2.83	****
Sildenafil	6.14	1.30	
Spiramycin	-4.73	4.48	
Spironolactone	-16.96	5.65	*
Sulfamethoxazole	-3.95	8.96	
Sulfasalazine	3.74	14.07	
Sulfinpyrazone	4.44	8.61	

Sulfisoxazole	5.50	1.92	
Sulindac	-14.91	6.34	
Tenoxicam	0.29	7.75	
Tetracycline hydrochloride	0.89	2.87	
Theophylline	-3.67	4.36	
Thioguanine	-57.74	8.26	****
Thiothixene	-82.55	1.35	****
Timolol Maleate	-1.46	0.83	
Tolbutamide	-8.82	6.19	
Tolfenamic Acid	-0.21	9.94	
Topiramate	6.44	11.94	
Trandolapril	1.72	4.39	
Tranexamic Acid	-4.15	10.88	
Tranylcypromine sulfate	1.35	7.49	
Trazodone Hydrochloride	3.24	4.79	
Trifluoperazine hydrochloride	-80.13	3.46	****
Trihexyphenidyl hydrochloride	-1.34	4.81	
Trimeprazine tartrate	-3.91	8.92	
Trimethoprim	-14.45	8.50	
Trimipramine Maleate	14.22	6.24	
Ursodiol	-4.24	6.78	
Valsartan Sodium	-5.47	1.29	
Vancomycin Hydrochloride	-3.59	1.64	
Venlafaxine	4.94	5.50	
Verapamil Hydrochloride	-5.94	4.07	
Yohimbine Hydrochloride	1.53	13.00	
Zidovudine [Azt]	-10.49	4.35	
Zolmitriptan	3.68	6.62	

Supplementary Methods

Chemical synthesis

Benzyl 2-acetamido-2,4-dideoxy-4-fluoro-3,6-di-O-pivaloyl-α-D-glucopyranoside (**12**): The alcohol **11** (3.3 g, 6.88 mmol) was dissolved in anhydrous CH_2Cl_2 (37 mL) in a Teflon flask, and the solution was cooled to 0 °C. Diethylaminosulfur trifluoride (DAST) (5.9 mL, 6.5 eq, 44.72 mmol) was added and the temperature of the reaction was allowed to rise to room temperature. After stirring for 17h, the mixture was diluted with ethyl acetate (150 mL), washed successively with a solution of $NaHCO_3$ (sat. 50 mL) and brine (10%, 50 mL), dried over anhydrous Na_2SO_4 and evaporated under reduced pressure. The crude product was purified by column chromatography on silica gel using a mixture of 10% EtOAc - toluene as the eluent to afford the desired compound **12** (1.9 g, yield: 57%). R_f = 0.54 (AcOEt : Hexane, 1 : 1 v/v). ^{1}H NMR (400 MHz, $CDCl_3$): δ 7.40 – 7.27 (m, 5H), 5.80 (d, $J_{H\text{-}2,NH}$ = 9.7 Hz, 1H, NH), 5.36 (ddd, $J_{H\text{-}3,H\text{-}4}$ = 8.9 Hz, $J_{H\text{-}2,H\text{-}3}$ = 10.9 Hz, $J_{H\text{-}3,F}$ = 13.8 Hz, 1H, H-3), 4.85 (dd, $J_{H\text{-}1,H\text{-}2}$ = 3.3 Hz, $J_{H\text{-}1,F}$ = 3.3 Hz, 1H, H-1), 4.73 (d, J = 11.9 Hz, 1H, Bn), 4.49 (d, J = 11.8 Hz, 1H, Bn), 4.46 (ddd, $J_{H\text{-}3,H\text{-}4}$ = 9.0 Hz, $J_{H\text{-}4,H\text{-}5}$ = 9.6 Hz, $J_{H\text{-}4,F}$ = 51.6 Hz, 1H, H-4), 4.38 (m, 1H, H-6a), 4.30 (dddd, $J_{H\text{-}1,H\text{-}2}$ = 3.6 Hz, $J_{H\text{-}2,H\text{-}3}$ = 10.7 Hz, $J_{H\text{-}2,NH}$ = 9.7 Hz, $J_{H\text{-}2,F}$ = 0.7 Hz, 1H, H-2), 4.20 (ddd, $J_{H\text{-}6a,H\text{-}6b}$ = 12.2 Hz, $J_{H\text{-}5,H\text{-}6b}$ = 5.2 Hz, $J_{H\text{-}6b,F}$ = 1.2 Hz, 1H, H-6b), 4.07 (m, 1H, H-5), 1.86 (s, 3H, Ac), 1.23 (s, 9H, Piv), 1.18 (s, 9H, Piv); ^{13}C NMR (100 MHz, $CDCl_3$): δ 178.7 (CO), 178.0 (CO), 169.8 (CO), 136.4 (Ph), 128.6 (× 2, Ph), 128.3 (Ph), 128.1 (× 2, Ph), 96.3 (C-1), 87.1 (d, $J_{C\text{-}4,F}$ = 186.9 Hz, C-4), 70.7 (d, $J_{C\text{-}3,F}$ = 18.2 Hz, C-3), 69.75 (Bn), 67.8 (d, $J_{C\text{-}5,F}$ = 18.2 Hz, C-5), 62.1 (C-6), 51.4 (d, $J_{C\text{-}2,F}$ = 7.0 Hz,

C-2), 38.9 (Piv), 38.9 (Piv), 27.2 (Piv), 26.93 (Piv), 23.0 (Ac); ESI HRMS (m/z): $[M + H]^+$ calcd. for $C_{25}H_{37}FNO_7$, 482.2549; found, 482.2538.

Benzyl 2-acetamido-2,4-dideoxy-4-fluoro-α-D-glucopyranoside (**13**): Compound **12** (3.0 g, 6.3 mmol) was dissolved in anhydrous methanol (45 mL), a solution of NaOMe in MeOH (1.5 M, 1.0 mL) was added, and the mixture was stirred at 50 °C overnight. After cooling down to room temperature, the mixture was neutralized with Amberlite IR-120 (H^+). The resin was filtered off and the mixture was evaporated under reduced pressure. The crude mixture was purified by column chromatography on silica gel using 3% MeOH – CH_2Cl_2 as an eluent to afford compound **13** (1.90 g, yield 97%). R_f = 0.54 (MeOH : CH_2Cl_2, 1 : 9 v/v); ^{1}H NMR (400 MHz, CD_3OD): δ 7.53 – 7.20 (m, 5H, Ph), 4.88 (dd, $J_{H\text{-}1,H\text{-}2}$ = 3.3 Hz, $J_{H\text{-}1,F}$ = 3.3 Hz, 1H, H-1), 4.76 (d, J = 12.1 Hz, 1H, Bn), 4.54 (d, J = 12.1 Hz, 1H, Bn), 4.33 (high order ddd, $J_{H\text{-}3,H\text{-}4}$ = 9.8 Hz, $J_{H\text{-}4,H\text{-}5}$ = 8.5 Hz, $J_{H\text{-}4,F}$ = 50.8 Hz, 1H, H-4), 4.03 – 3.90 (m, 2H, H-2 + H-3), 3.87 – 3.77 (m, 2H, H-5 + H-6a), 3.73 (ddd, $J_{H\text{-}5,H\text{-}6b}$ = 5.0 Hz, $J_{H\text{-}6a,H\text{-}6b}$ = 12.2 Hz, $J_{H\text{-}6b,F}$ = 1.7 Hz, 1H, H-6b), 1.97 (s, 3H, Ac); ^{13}C NMR (100 MHz, CD_3OD): δ 172.2 (CO), 137.4 (Ph), 128.1 (Ph), 127.9 (Ph), 127.6 (Ph), 96.0 (C-1), 90.0 (d, $J_{C\text{-}4,F}$ = 180.1 Hz, C-4), 70.2 (d, $J_{C\text{-}5,F}$ = 24.4 Hz, C-5), 69.2 (d, $J_{C\text{-}3,F}$ = 19.0 Hz, C-3), 69.1 (Bn), 60.3 (C-6), 53.6 (d, $J_{C\text{-}2,F}$ = 8.3 Hz, C-2), 21.1 (Ac); ESI HRMS (m/z): $[M+H]^+$ calcd. for $C_{15}H_{21}FNO_5$, 314.1398; found, 314.1375.

Benzyl 2-acetamido-3,6-di-O-acetyl-2,4-dideoxy-4-fluoro-α-D-glucopyranoside (**14**): Compound **13** (1.8 g, 5.7 mmol) was dissolved in pyridine (15 mL), and acetic anhydride (10 mL) was added. After stirring the reaction for 3h at room temperature, the solution

was concentrated under reduced pressure to afford a mixture which was purified by column chromatography on silica gel using a mixture of 40% EtOAc - hexane as the eluent to provide compound **14** (2.2 g, yield: 96%). R_f = 0.16 (AcOEt : Hexane, 1 : 1 v/v); ^{1}H NMR (400 MHz, $CDCl_3$): δ 7.43 – 7.18 (m, 5H, Ph), 5.82 (d, $J_{H\text{-}2,NH}$ = 9.5 Hz, 1H, NH), 5.34 (ddd, $J_{H\text{-}2,H\text{-}3}$ = 10.9 Hz, $J_{H\text{-}3,H\text{-}4}$ = 8.9 Hz, $J_{H\text{-}3,F}$ = 14.2 Hz, 1H, H-3), 4.88 (dd, $J_{H\text{-}1,H\text{-}2}$ = 3.4 Hz, $J_{H\text{-}1,F}$ = 3.4 Hz, 1H, H-1), 4.70 (d, J = 11.8 Hz, 1H, Bn), 4.50 (ddd, $J_{H\text{-}4,H\text{-}5}$ = 9.2, Hz, $J_{H\text{-}3,H\text{-}4}$ = 9.5, Hz, $J_{H\text{-}4,F}$ = 50.9 Hz, 1H, H-4), 4.50 (d, J = 11.8 Hz, 1H, Bn), 4.35 – 4.19 (m, 3H, H-6a + H-6b + H-2), 4.00 (m, 1H, H-5), 2.09 (s, 3H), 2.06 (s, 3H), 1.88 (s, 3H); ^{13}C NMR (100 MHz, $CDCl_3$): δ 171.1 (CO), 170.5 (CO), 170.0 (CO), 136.4 (Ph), 128.7 (× 2, Ph), 128.4 (Ph), 128.2 (× 2, Ph), 96.4 (d, $J_{C\text{-}1,F}$ = ~1 Hz, C-1), 86.6 (d, $J_{C\text{-}4,F}$ = 186.4 Hz, C-4), 71.1 (d, $J_{C\text{-}3,F}$ = 18.4 Hz, C-3), 70.21 (CH$_2$Ph), 67.4 (d, $J_{C\text{-}5,F}$ = 23.4 Hz, C-5), 61.9 (C-6), 51.6 (d, $J_{C\text{-}2,F}$ = 7.0 Hz, C-2), 23.0 (Ac), 20.7 (Ac), 20.7 (Ac); ESI HRMS (m/z): $[M+H]^+$ calcd. for $C_{19}H_{25}FNO_7$, 398.1610; found, 398.1597.

2-Acetamido-2,4-dideoxy-4-fluoro- α,β-D-glucopyranose (**3**): Compound **13** (50 mg 0.16 mmol) was hydrogenated in MeOH (3 mL) in the presence of 20% $Pd(OH)_2$-C (~10 mg) overnight as above to afford the desired compound **3**, which was not purified further. Compound **3** was obtained as an anomeric mixture (α/β , 84.2/15.8, 34 mg, 95% yield). R_f = 0.23 (MeOH : CH_2Cl_2, 1 : 9 v/v); ^{1}H NMR (600 MHz, CD_3OD) for α-anomer: δ 5.11 (dd, $J_{H\text{-}1,H\text{-}2}$ = 3.0 Hz, $J_{H\text{-}1,F}$ = 3.0 Hz,1H, H-1), 4.31 (ddd, $J_{H\text{-}3,H\text{-}4}$ = 9.5 Hz, $J_{H\text{-}4,H\text{-}5}$ = 8.9 Hz, $J_{H\text{-}4,F}$ = 51.2 Hz, 1H, H-4), 4.02 – 3.94 (m, 2H, H-3 + H-5), 3.90 (dd, $J_{H\text{-}1,H\text{-}2}$ = 3.2 Hz, $J_{H\text{-}2,H\text{-}3}$ = 10.8 Hz, 1H, H-2), 3.77 (m, 1H, H-6a), 3.72 (ddd, $J_{H\text{-}6a,H\text{-}6b}$ = 12.1 Hz, $J_{5,H\text{-}6b}$ = 4.3 Hz, $J_{H\text{-}6b,F}$ = 1.0

Hz, 1H, H-6b), 2.04 (s, 3H, Ac); ^{13}C NMR (150 MHz, CD_3OD): δ 172.7 (CO), 90.9 (C-1), 90.0 (d, $J_{C\text{-}4,F}$ = 180.1 Hz, C-4), 69.2 (d, $J_{C\text{-}5,F}$ = 24.1 Hz, C-5), 69.2 (d, $J_{C\text{-}3,F}$ = 18.8 Hz, C-3), 60.4 (C-6), 54.3 (d, $J_{C\text{-}2,F}$ = 7.9 Hz, C-2), 21.0 (Ac); Selective ^{1}H NMR (600 MHz, CD_3OD) for β-anomer: δ 4.68 (d, $J_{H\text{-}1,H\text{-}2}$ = 8.0 Hz, 1H, H-1), 4.27 (overlapped, 1H, H-4), 3.78 (m, 1H, H-3), 3.97 (overlapped, 1H, H-2), 3.83 (m, 1H, H-6a), 3.74 (overlapped, 1H, H-6b), 3.49 (m, 1H, H-5), 2.04 (s, 3H, Ac); ^{13}C NMR (150 MHz, CD_3OD): δ 172.7 (CO), 95.4 (C-1), 89.6 (d, $J_{C\text{-}4,F}$ = 180.1 Hz, C-4), 74.0 (d, $J_{C\text{-}5,F}$ = 24.3 Hz, C-5), 72.2 (d, $J_{C\text{-}3,F}$ = 18.6 Hz, C-3), 60.4 (C-6), 57.3 (d, $J_{C\text{-}2,F}$ = 8.6 Hz, C-2), 21.3 (Ac); ESI HRMS (m/z): $[M + Na]^+$ calcd. for $C_8H_{14}FNO_5Na$, 246.0748; found, 246.0750.

2-Acetamido-1,3,6-tri-O-acetyl-2,4-dideoxy-4-fluoro-α,β-D-glucopyranose (**4**): The benzyl glycoside **14** (1.53 g, 3.9 mmol) was dissolved in MeOH (16 mL), and CH_2Cl_2 (4 mL) was added along with H_2O (4 drops); 20% $Pd(OH)_2$ on charcoal (50 mg) was added, and the reaction mixture was purged with hydrogen gas and stirred in a hydrogen atmosphere for 24h. The catalyst was removed by filtration and the solution was concentrated under reduced pressure. The obtained mixture was redissolved in pyridine (10 mL), and acetic anhydride (8 mL) was added; the reaction was stirred for 1h at room temperature. The solution was concentrated under high vacuum, and the obtained mixture was purified by column chromatography on silica gel using a mixture of 50% EtOAc - hexane as eluent to afford the desired compound **4** as an anomeric mixture (α/β, 86.4/13.6, 1.29 g, yield: 96%). R_f = 0.20 (AcOEt : Hexane, 7 : 3 v/v); ^{1}H NMR (400 MHz, $CDCl_3$) for α-anomer: δ 6.14 (dd, $J_{H\text{-}1,H\text{-}2}$ = 3.3 Hz, $J_{H\text{-}1,F}$ = 3.5 Hz, 1H, H-1), 5.62 (d, $J_{H\text{-}2,NH}$ = 9.2 Hz, 1H, NH), 5.36 (ddd, $J_{H\text{-}2,H\text{-}3}$ = 11.1 Hz, $J_{H\text{-}3,H\text{-}4}$ = 9.0 Hz, $J_{H\text{-}3,F}$ = 13.9 Hz, 1H, H-3), 4.61 (ddd, $J_{H\text{-}3,H\text{-}4}$ = 9.1

Hz, $J_{H-4,H-5}$ = 9.9 Hz, $J_{H-4,F}$ = 50.5 Hz, 1H, H-4), 4.42 (dddd, $J_{H-1,H-2}$ = 3.4 Hz, $J_{H-2,H-3}$ = 11.1 Hz, $J_{H-2,NH}$ = 9.0 Hz, $J_{H-2,F}$ = 1.0 Hz, 1H, H-2), 4.37 (ddd, $J_{H-5,H-6a}$ = 2.1 Hz, $J_{H-6a,H-6b}$ = 12.3 Hz, $J_{H-6a,F}$ = 2.1 Hz, 1H, H-6a), 4.27 (ddd, $J_{H-5,H-6a}$ = 4.2 Hz, $J_{H-6a,H-6b}$ = 12.4 Hz, $J_{H-6b,F}$ = 1.2 Hz, 1H, H-6b), 4.05 (m, 1H, H-5), 2.21 (s, 3H, Ac), 2.15 (s, 3H, Ac), 2.11 (s, 3H, Ac), 1.96 (s, 3H, Ac); ^{13}C NMR (100 MHz, CD_3OD): δ 171.5 (CO), 170.5 (CO), 170.2 (CO), 168.6 (CO), 90.4 (d, $J_{C-1,F}$ = 0.9 Hz, C-1), 86.1 (d, $J_{C-4,F}$ = 186.7 Hz, C-4), 70.5 (d, $J_{C-3,F}$ = 18.8 Hz, C-3), 69.1 (d, $J_{C-5,F}$ = 23.8 Hz, C-5), 61.6 (C-6), 50.7 (d, $J_{C-2,F}$ = 7.2 Hz, C-2), 22.9 (Ac), 20.8 (Ac), 20.76 (Ac), 20.6 (Ac); Selected ^{1}H NMR (400 MHz, $CDCl_3$) for β-anomer: δ 5.61 (d, $J_{H-1,H-2}$ = 8.8 Hz, 1H, H-1), 5.55 (d, d, $J_{H-2,NH}$ = 9.6 Hz, 1H, NH), 5.21 (ddd, $J_{H-2,H-3}$ = 10.9 Hz, $J_{H-3,H-4}$ = 8.8 Hz, $J_{H-3,F}$ = 14.1 Hz, 1H, H-3), 4.56 (ddd, ddd, $J_{H-3,H-4}$ = 8.9 Hz, $J_{H-4,H-5}$ = 9.6 Hz, $J_{H-4,F}$ = 50.4 Hz, 1H, H-4), 3.84 (m, 1H, H-5), 2.13 (s, 3H, Ac), 2.12 (s, 3H, Ac), 2.05 (s, 3H, Ac), 1.95 (s, 3H, Ac); Selected ^{13}C NMR (100 MHz, $CDCl_3$): δ 170.9 (CO), 170.2 (CO), 169.4 (CO), 168.6 (CO), 92.4 (C-1), 86.2 (d, $J_{C-4,F}$ = 187.4 Hz, C-4), 72.4 (d, $J_{C-3,F}$ = 18.9 Hz, C-3), 72.1 (d, $J_{C-5,F}$ = 24.3 Hz, C-5), 61.8 (C-6), 52.5 (d, $J_{C-2,F}$ = 7.0 Hz, C-2), 23.1 (Ac), 20.8 (Ac), 20.76 (Ac), 20.7 (Ac); ESI HRMS (m/z): [M + Na]$^+$ calcd. for $C_{14}H_{20}FNO_8Na$, 372.1065; found, 372.1054.

2-Acetamido-1,3,6-tri-O-acetyl-2,4-dideoxy-4-fluoro-α,β-D-glucopyranose (**5**): The benzyl glycoside **14** (1.0 g, 3.9 mmol) was dissolved in a mixture of MeOH (15 mL), CH_2Cl_2 (3 mL) and H_2O (4 drops). The mixture was hydrogenated with 20% $Pd(OH)_2$ on charcoal (50 mg) as above. After 24h, the catalyst was removed by filtration and the solution was concentrated under reduced pressure. The solid mixture was purified by column chromatography on silica gel using a mixture of 40% EtOAc-hexane as eluent to afford

the desired compound **5** as an inseparable anomeric mixture (α/β, 91.1/8.9, 750 mg, yield: 97%). R_f = 0.11 (AcOEt : Hexane, 7 : 3 v/v); ^{1}H NMR (400 MHz, CD_3OD) for α-anomer: δ 5.43 (ddd, $J_{H-2,H-3}$ = 10.8 Hz, $J_{H-3,H-4}$ = 8.8 Hz, $J_{H-3,F}$ = 14.2 Hz, 1H, H-3), 5.08 (dd, $J_{H-1,H-2}$ = 3.4 Hz, $J_{H-1,F}$ = 3.4 Hz, 1H, H-1), 4.49 (ddd, $J_{H-3,H-4}$ = 9.0 Hz, $J_{H-4,H-5}$ = 9.5 Hz, $J_{H-4,F}$ = 51.1 Hz, 1H, H-4), 4.40 (m, 1H, H-6a), 4.33 – 4.20 (m, 2H, H-5 + H-6b), 4.16 (ddd, $J_{H-1,H-2}$ = 3.4 Hz, $J_{H-2,H-3}$ = 10.8 Hz, $J_{H-2,F}$ = 0.8 Hz, 1H, H-2), 2.09 (s, 3H, Ac), 2.07 (s, 3H, Ac), 1.96 (s, 3H, Ac); ^{13}C NMR (100 MHz, CD_3OD) for α-anomer: δ 172.1 (CO), 171.1 (CO), 170.7 (CO), 91.2 (d, $J_{C-1,F}$ = 1.2 Hz, C-1), 87.7 (d, $J_{C-4,F}$ = 185.4 Hz, C-4), 71.0 (d, $J_{C-3,F}$ = 18.4 Hz, C-3), 66.6 (d, $J_{C-5,F}$ = 23.1 Hz, C-5), 62.3 (C-6), 52.0 (d, $J_{C-2,F}$ = 7.1 Hz, C-2), 21.07 (Ac), 19.31 (Ac), 19.21 (Ac). Selected ^{1}H NMR (400 MHz, CD_3OD) for β-anomer: δ 5.31 (ddd, $J_{H-2,H-3}$ = 10.6 Hz, $J_{H-3,H-4}$ = 8.8 Hz, $J_{H-3,F}$ = 14.9 Hz, 1H, H-3), 4.83 (d, $J_{H-1,H-2}$ = 8.4 Hz, 1H, H-1), 3.86 (m, 1H, H-5), 3.81 (ddd, $J_{H-1,H-2}$ = 8.3 Hz, $J_{H-2,H-3}$ = 10.4 Hz, $J_{H-2,F}$ = 0.8 Hz, 1H, H-2), 2.09 (s, 3H, Ac), 2.07 (s, 3H, Ac), 1.96 (s, 3H, Ac); Selected ^{13}C NMR (100 MHz, CD_3OD) for β-anomer: δ 172.2 (CO), 171.0 (CO), 170.4 (CO), 95.0 (d, $J_{C-1,F}$ = 1.3 Hz, C-1), 87.4 (d, $J_{C-4,F}$ = 185.9 Hz, C-4), 72.8 (d, $J_{C-3,F}$ = 18.8 Hz, C-3), 71.0 (d, $J_{C-5,F}$ = 23.5 Hz, C-5), 62.3 (C-6), 55.2 (d, $J_{C-2,F}$ = 7.6 Hz, C-2), 21.36 (Ac), 19.31 (Ac), 19.24 (Ac); ESI HRMS (m/z): $[M + Na]^+$ calcd. for $C_{12}H_{18}FNO_7Na$, 330.0960; found. 330.0958.

2-Acetamido-3,6-di-O-acetyl-2,4-dideoxy-4-fluoro-1-O-propanoyl-α,β-D-glucopyranose (**6**): The hemiacetal **5** (70 mg, 0.23 mmol) was dissolved in a mixture of anhydrous CH_2Cl_2 (1.5 mL) and pyridine (1.0 mL), and propionic anhydride (58 μL, 0.46 mmol) was added. The mixture was stirred at room temperature for 4h and quenched with MeOH (50 μL). The solution was concentrated under reduced pressure, and the residue was

purified by column chromatography on silica gel using a mixture of 30% EtOAc – hexane as the eluent to afford the desired compound **6** as an anomeric mixture (*α*/*β*, 95.5/4.5, 69.5 mg, 84% yield). R_f = 0.30 (AcOEt : Hexane, 7 : 3 v/v); ^{1}H NMR (400 MHz, CD_3COCD_3) for *α*-anomer: *δ* 7.16 (d, $J_{NH,H-2}$ = 9.2 Hz, 1H, NH), 6.10 (dd, $J_{H-1,H-2}$ = 3.3 Hz, $J_{H-1,F}$ = 3.3 Hz, 1H, H-1), 5.38 (ddd, $J_{H-2,H-3}$ = 11.1 Hz, $J_{H-3,H-4}$ = 9.0 Hz, $J_{H-3,F}$ = 13.8 Hz, 1H, H-3), 4.67 (ddd, $J_{H-3, H-4}$ = 9.3 Hz, $J_{H-4, H-5}$ = 9.3 Hz, $J_{H-4,F}$ = 50.7 Hz, 1H, H-4), 4.39 (dddd, $J_{H-1, H-2}$ = 3.6 Hz, $J_{H-2,H-3}$ = 11.0 Hz, $J_{H-2,NH}$ = 9.6 Hz, $J_{H-2,F}$ = 1.0 Hz, 1H, H-2), 4.33 (m, 1H, H-6a), 4.26 – 4.17 (m, 2H, H-5 + H-6b), 2.51 (q, *J* = 7.5 Hz, 2H, CH_3CH_2CO), 2.05 (s, 6H, 2 × Ac), 1.84 (s, 3H, Ac), 1.12 (t, *J* = 7.5 Hz, 3H, CH_3CH_2CO); ^{13}C NMR (100 MHz, CD_3COCD_3) for *α*-anomer: *δ* 172.2 (CO), 169.9 (CO), 169.8 (CO), 90.0 (d, $J_{C-1,F}$ = 1.5 Hz, C-1), 87.1 (d, $J_{C-4,F}$ = 184.6 Hz, C-4), 70.2 (d, $J_{C-3,F}$ = 18.5 Hz, C-3), 69.2 (d, $J_{C-5,F}$ = 23.4 Hz, C-5), 61.5 (C-6), 50.4 (d, $J_{C-2,F}$ = 7.6 Hz, C-2), 26.8 (CH_3CH_2CO), 21.7 (Ac), 19.8 (Ac), 19.7 (Ac), 8.2 (CH_3CH_2CO); Selective ^{1}H NMR (400 MHz, CD_3COCD_3) for *β*-anomer: *δ* 7.16 (overlapped, 1H, NH), 5.87 (d, $J_{H-1,H-2}$ = 8.6 Hz, 1H, H-1), 5.41 (overlapped, 1H, H-3), 4.59 (ddd, $J_{H-3, H-4}$ = 9.7 Hz, $J_{H-4, H-5}$ = 8.9 Hz, $J_{H-4,F}$ = 50.7 Hz, 1H, H-4), 4.01 (m, 1H, H-5), 2.35 (q, *J* = 7.5 Hz, 2H, CH_3CH_2CO), 2.06 (s, 3H, Ac), 2.04 (s, 3H, Ac), 1.83 (s, 3H, Ac), 1.07 (t, *J* = 7.5 Hz, 3H, CH_3CH_2CO); ESI HRMS (m/z): $[M + Na]^+$ calcd. for $C_{15}H_{22}FNO_8Na$, 386.1222; found 386.1215.

Benzyl 2-acetamido-2-deoxy-3,6-di-O-pivaloyl-α-D-glucopyranoside (**10**): A solution of compound **9** (40 g, 0.13 mol) in anhydrous dichloromethane (400 mL) and pyridine (100 mL) was cooled to 0 °C, and pivaloyl chloride (48 mL, 0.39 mol) was added dropwise.

After stirring the mixture for 2h, the reaction was quenched with H_2O (20 mL). The solution was evaporated, and the mixture was dissolved in ethyl acetate (200 mL), washed with H_2O (2 × 200 mL), dried over anhydrous Na_2SO_4 and evaporated. The mixture was purified by column chromatography on silica gel using a mixture of 15% ethyl acetate - hexane as eluent to afford the desired compound **10** (34.5 g, 56% yield). R_f = 0.5 (AcOEt : Hexane, 1 : 1 v/v); ^{1}H NMR (400 MHz, $CDCl_3$): δ 7.43 – 7.30 (m, 5H), 5.75 (d, $J_{NH,H\text{-}2}$ = 9.5 Hz, 1H, NH), 5.12 (dd, $J_{H\text{-}2,H\text{-}3}$ = 10.8 Hz, $J_{H\text{-}3,H\text{-}4}$ = 9.3 Hz, 1H, H-3), 4.90 (d, $J_{H\text{-}1,H\text{-}2}$ = 3.7 Hz, 1H, H-1), 4.75 (d, J = 11.8 Hz, 1H, Bn), 4.50 (d, J = 11.8 Hz, 1H, Bn), 4.40 (dd, $J_{H\text{-}5,H\text{-}6a}$ = 4.8 Hz, $J_{H\text{-}6a,H\text{-}6b}$ = 12.2 Hz, 1H, H-6a), 4.32 (m, 2H, H-6b + H-2), 3.90 (ddd, $J_{H\text{-}4,H\text{-}5}$ = 9.9 Hz, $J_{H\text{-}5,H\text{-}6a}$ = 4.7 Hz, $J_{H\text{-}5,H\text{-}6b}$ = 2.2 Hz, 1H, H-5), 3.56 (dd, $J_{H\text{-}4,H\text{-}5}$ = 9.6 Hz, $J_{H\text{-}4,H\text{-}5}$ = 9.6 Hz, 1H, H-4), 3.04 (br s, 1H, OH-4), 1.89 (s, 3H, Ac), 1.28 (s, 9H, Piv), 1.21 (s, 9H, Piv); ^{13}C NMR (100 MHz, $CDCl_3$): δ 179.9 (CO), 179.2 (CO), 169.8 (CO), 136.7 (Ph), 128.7 (Ph), 128.3 (Ph), 128.1 (Ph), 96.1 (C-1), 73.4 (C-3), 70.7 (C-5), 69.6 ($PhCH_2$), 69.2 (C-4), 63.1 (C-6), 51.4 (C-2), 39.0 (× 2, Piv), 27.3 (Piv), 27.0 (Piv), 23.1 (Ac); ESI HRMS (m/z): $[M + H]^+$ calcd. for $C_{25}H_{38}NO_8$, 480.2592; found, 480.2573.

Benzyl 2-acetamido-2-deoxy-3,6-di-O-pivaloyl-α-D-galactopyranoside (**11**): A solution of compound **10** (3.8 g, 7.9 mmol) in a mixture of anhydrous pyridine (12 mL) and dichloromethane (12 mL) was cooled to -10 °C, trifluoromethanesulfonic anhydride (2.66 mL, 15.8mmol) was added dropwise. After stirring the mixture for 1 h, H_2O (2 mL) was added to quench the reaction. The mixture was dissolved in EtOAc (40 mL) and washed successively with 1 N HCl (40 mL), saturated $NaHCO_3$ (40 mL) and brine (40 mL), and

dried over anhydrous Na_2SO_4. The organic solution was concentrated under reduced pressure. The crude product was dissolved in DMF (25 mL); sodium nitrite (1.72 g, 24.9 mmol) and water (5 drops) were added to crude solution. After stirring overnight at room temperature, the mixture was concentrated under reduced pressure, and the residue was diluted with ethyl acetate (40 mL), washed with H_2O (2 × 40 mL), dried over anhydrous Na_2SO_4 and concentrated under reduced pressure. The residue was purified by column chromatography on silica gel using a mixture of 20% ethyl acetate - hexane as eluent to afford the desired compound **11** (2.1 g, 55% yield). R_f = 0.31 (AcOEt : Hexane, 1 : 1 v/v); ^{1}H NMR (400 MHz, $CDCl_3$): δ 7.42 – 7.29 (m, 5H, Ph), 5.64 (d, $J_{H\text{-}2,NH}$ = 9.9 Hz, 1H, NH), 5.13 (dd, $J_{H\text{-}2,H\text{-}3}$ = 11.2 Hz, $J_{H\text{-}3,H\text{-}4}$ = 3.0 Hz, 1H, H-3), 4.95 (d, $J_{H\text{-}1,H\text{-}2}$ = 3.7 Hz, 1H, H-1), 4.76 (d, J = 11.7 Hz, Bn), 4.73 (ddd, $J_{H\text{-}1,H\text{-}2}$ = 3.8 Hz, $J_{H\text{-}2,NH}$ = 9.9 Hz, $J_{H\text{-}2,H\text{-}3}$ = 11.2 Hz, H-2), 4.49 (d, J = 11.7 Hz, 1H, Bn), 4.36 (dd, $J_{H\text{-}5,H\text{-}6a}$ = 5.1 Hz, $J_{H\text{-}6a,H\text{-}6b}$ = 11.5 Hz, 1H, H-6a), 4.28 (dd, $J_{H\text{-}5,H\text{-}6b}$ = 7.1 Hz, $J_{H\text{-}6a,H\text{-}6b}$ = 11.6 Hz, 1H, H-6b), 4.12 (ddd, $J_{H\text{-}4,H\text{-}5}$ = 1.0 Hz, $J_{H\text{-}5,H\text{-}6a}$ = 5.1 Hz, $J_{H\text{-}5,H\text{-}6b}$ = 7.0 Hz, 1H, H-5), 4.1 (dd, $J_{H\text{-}4,H\text{-}5}$ = 1.0 Hz, $J_{H\text{-}3,H\text{-}4}$ = 3.0 Hz, 1H, H-4), 2.58 (br, 1H, OH-4), 1.90 (s, 3H, Ac), 1.24 (s, 9H, Piv), 1.21 (s, 9H, Piv); ^{13}C NMR (100 MHz, $CDCl_3$): δ 178.5 (CO), 178.3 (CO), 169.8 (CO), 136.8 (Ph), 128.7 (Ph), 128.2 (Ph), 128.1 (Ph), 96.9 (C-1), 70.7 (C-3), 69.50 (Ph*C*H$_2$), 68.6 (C-5), 67.7 (C-4), 63.4 (C-6), 47.3 (C-2), 39.0 (Piv), 38.8 (Piv), 27.2 (Piv), 27.1 (Piv), 23.2 (Ac); ESI HRMS (m/z): $[M+H]^+$ calcd. for $C_{25}H_{38}NO_8$, 480.2592; found, 480.2581.

Benzyl 2-acetamido-2-deoxy-4-O-methyl-3,6-di-O-pivaloyl-α-D-glucopyranoside (**15**): To a solution of compound **10** (300 mg, 0.61 mmol) in anhydrous DMF (3.5 mL), was added

NaH (50 mg, 2.1 mmol), and the solution was stirred for 30 min at room temperature and then CH_3I (62 μL, 6.3 mmol) was added. After stirring the solution for 1h at room temperature, the reaction was quenched with methanol (~1 mL). The crude mixture was purified by column chromatography on silica gel using a gradient of 10→30% EtOAc–Hexane as the eluent to yield compound **15** (122 mg, 38% yield). R_f = 0.54 (EtOAc : Hexane, 1 : 1 v/v); $[\alpha]_D^{25}$: + 65.9 (*c* 0.43, $CHCl_3$); ^1H NMR (400 MHz, $CDCl_3$): δ 7.40 – 7.28 (m, 5H, Ph), 5.67 (d, $J_{H-2,NH}$ = 9.7 Hz, 1H, NH), 5.25 (dd, $J_{H-2,H-3}$ = 10.9 Hz, $J_{H-3,H-4}$ = 9.3 Hz, 1H, H-3), 4.85 (d, $J_{H-1,H-2}$ = 3.6 Hz, 1H, H-1), 4.72 (d, J = 11.9 Hz, 1H, Bn), 4.47 (d, J = 11.9 Hz, 1H, Bn), 4.36 (dd, $J_{H-6a,H-6b}$ = 12.0 Hz, $J_{H-5,H-6a}$ = 2.0 Hz, 1H, H-6a), 4.27 (ddd, $J_{H-1,H-2}$ = 3.7 Hz, $J_{H-2,NH}$ = 9.9 Hz, $J_{H-2,H-3}$ = 10.9 Hz, 1H, H-2), 4.21 (dd, $J_{H-5,H-6b}$ = 4.6 Hz $J_{H-6a,H-6b}$ = 12.1 Hz, 1H, H-6b), 3.88 (ddd, $J_{H-4,H-5}$ = 9.9 Hz, $J_{H-5,H-6b}$ = 4.5 Hz, $J_{H-5,H-6a}$ = 2.0 Hz, 1H, H-5), 3.43 (s, 3H, OMe), 3.36 (dd, $J_{H-3,H-4}$ = 9.6 Hz, $J_{H-4,H-5}$ = 9.6 Hz, 1H, H-4), 1.87 (s, 3H, Ac), 1.26 (s, 9H, Piv), 1.21 (s, 9H, Piv); ^{13}C NMR (100 MHz, $CDCl_3$): δ 178.8 (CO), 178.1 (CO), 169.6 (CO), 136.7 (Ph), 128.5 (Ph), 128.1 (Ph), 127.9 (Ph), 96.5 (C-1), 77.9 (C-4), 72.8 (C-3), 69.5 (Bn), 69.4 (C-5), 62.6 (C-6), 60.5 (OMe), 52.0 (C-2), 38.84 (Piv), 38.8 (Piv), 27.2 (Piv), 27.0 (Piv), 23.0 (Ac); ESI HRMS (m/z): $[M + H]^+$ calcd. for $C_{26}H_{40}NO_8$, 494.2748; found, 494.2735.

Benzyl 2-acetamido-2-deoxy-4-O-methyl-α-D-glucopyranoside (**16**): To a solution of 4-*O*-methylated compound **15** (122 mg, 0.25 mmol) in anhydrous methanol (3 mL), was added a solution of NaOMe in MeOH (1.5 M, 0.1 mL), and the solution was stirred at 55 °C for 2h and then quenched by adding dry ice. The solution was concentrated under reduced pressure and the crude mixture was purified by column chromatography on

silica gel using a gradient of 2→ 5% methanol– CH_2Cl_2 as the eluent to yield compound **16** (74 mg, 91% yield). R_f = 0.46 (Methanol : CH_2Cl_2, 1 : 10 v/v); $[\alpha]_D^{25}$: + 75.6 (*c* 0.43, $CHCl_3$); ^{1}H NMR (400 MHz, CD_3OD): δ 7.42 – 7.21 (m, 5H, Ph), 4.84 (d, $J_{H\text{-}1,H\text{-}2}$ = 3.6 Hz, 1H, H-1), 4.72 (d, J = 12.1 Hz, 1H, Bn), 4.49 (d, J = 12.1 Hz, 1H, Bn), 3.91 (dd, $J_{H\text{-}2,H\text{-}3}$ = 10.8 Hz, $J_{H\text{-}1,H\text{-}2}$ = 3.6 Hz, 1H, H-2), 3.79 (dd, $J_{H\text{-}2,H\text{-}3}$ = 10.6 Hz, $J_{H\text{-}3,H\text{-}4}$ = 8.7 Hz, 1H, H-3), 3.77 (dd, $J_{H\text{-}5,H\text{-}6b}$ = 2.0 Hz, $J_{H\text{-}6a,H\text{-}6b}$ = 11.7 Hz, 1H, H-6a), 3.69 (dd, $J_{H\text{-}6a,H\text{-}6b}$ = 11.6 Hz, $J_{H\text{-}5,H\text{-}6b}$ = 4.7 Hz, 1H, H-6b), 3.60 (ddd, $J_{H\text{-}5,H\text{-}6a}$ = 2.2 Hz, $J_{H\text{-}5,H\text{-}6b}$ = 4.6 Hz, $J_{H\text{-}4,H\text{-}5}$ = 9.6 Hz, 1H, H-5), 3.58 (s, 3H, OMe), 3.16 (dd, $J_{H\text{-}4,H\text{-}5}$ = 9.8 Hz, $J_{H\text{-}3,H\text{-}4}$ = 8.8 Hz, 1H, H-4), 1.94 (s, 3H, Ac); ^{13}C NMR (100 MHz, CD_3OD): δ 172.1 (CO), 137.5 (Ph), 127.9 (Ph), 127.7 (Ph), 127.3 (Ph), 95.9 (C-1), 80.3 (C-4), 71.7 (C-5), 71.3 (C-3), 68.9 (Bn), 60.7 (C-6), 59.6 (OMe), 54.0 (C-2), 21.0 (Ac); ESI HRMS (m/z): $[M + Na]^+$ calcd. for $C_{16}H_{23}NO_6Na$, 348.1418; found, 348.1413.

Benzyl 2-acetamido-3,6-di-O-acetyl-2-deoxy-4-O-methyl-α-D-glucopyranoside (**17**): To a solution of compound **16** (180 mg, 0.55 mmol) in dry pyridine (3 mL), was added acetic anhydride (0.5 mL), and the solution was stirred at 55 °C for 1h. The solution was concentrated under reduced pressure. The obtained crude mixture was purified by column chromatography on silica gel using a mixture of 2% methanol– CH_2Cl_2 as the eluent to yield compound **17** (225 mg, 99% yield). R_f = 0.76 (Methanol : CH_2Cl_2, 5 : 95 v/v); $[\alpha]_D^{25}$: + 79.3 (*c* 0.43, $CHCl_3$); ^{1}H NMR (400 MHz, $CDCl_3$): δ 7.40 – 7.28 (m, 5H, Ph), 5.72 (d, $J_{H\text{-}2,NH}$ = 9.6 Hz, 1H, NH), 5.20 (dd, $J_{H\text{-}2,H\text{-}3}$ = 10.8 Hz, $J_{H\text{-}3,H\text{-}4}$ = 9.2 Hz, 1H, H-3), 4.87 (d, $J_{H\text{-}1,H\text{-}2}$ = 3.7 Hz, 1H, H-1), 4.70 (d, J = 11.8 Hz, 1H, Bn), 4.48 (d, J = 11.8 Hz, 1H, Bn), 4.30 (dd, $J_{H\text{-}6a,H\text{-}6b}$ = 12.0 Hz, $J_{H\text{-}5,H\text{-}6a}$ = 2.4 Hz, 1H, H-6a), 4.25 (dd, $J_{H\text{-}6a,H\text{-}6b}$ = 11.9 Hz,

$J_{H-5,H-6b}$ = 4.1 Hz, 1H, H-6b), 4.24 (ddd, $J_{H-2,H-3}$ = 10.8 Hz, $J_{H-2,NH}$ = 9.6 Hz, $J_{H-1,H-2}$ = 3.7 Hz, 1H, H-2), 3.83 (ddd, $J_{H-4,H-5}$ = 10.0 Hz, $J_{H-5,H-6b}$ = 3.9 Hz, $J_{H-5,H-6a}$ = 2.6 Hz, 1H, H-5), 3.44 (s, 3H, OMe), 3.38 (dd, $J_{H-3,H-4}$ = 9.6 Hz, $J_{H-4,H-5}$ = 9.6 Hz, 1H, H-4), 2.12 (s, 3H, Ac), 2.08 (s, 3H, Ac), 1.89 (s, 3H, Ac); ^{13}C NMR (100 MHz, $CDCl_3$): δ 171.2 (CO), 170.6 (CO), 169.9 (CO), 136.6 (Ph), 128.5 (Ph), 128.2 (Ph), 128.1 (CO), 96.5 (C-1), 77.3 (C-4), 73.7 (C-3), 69.9 (Bn), 69.1 (C-5), 62.6 (C-6), 60.4 (OMe), 52.0 (C-2), 23.1 (Ac), 20.9 (Ac), 20.7 (Ac); ESI HRMS (m/z): $[M + H]^+$ calcd. for $C_{20}H_{28}NO_8$, 410.1809; found, 410.1803.

2-Acetamido-1,3,6-tri-O-acetyl-2-deoxy-4-O-methyl-α/β-D-glucopyranose (**7**): Compound **17** (225 mg, 0.55 mmol) was dissolved in a mixture of methanol (10 mL), CH_2Cl_2 (2 mL) and water (10 drops), and 10% Pd-C (100 mg) was added. The mixture was stirred at room temperature under hydrogen atmosphere overnight. The catalyst was filtered off and the solution was concentrated under reduced pressure. The residue was acetylated using a mixture of acetic anhydride (0.5 mL) and pyridine (3.0 mL) at room temperature for 1h. After removing the solvents from the reaction, the crude mixture was purified by column chromatography on silica gel using a gradient of 0.5→1.5% methanol–CH_2Cl_2 as the eluent to afford compound **7** as an anomeric mixture (*α*/*β*, 95.4/4.6, 61 mg, 31% yield). R_f = 0.39 (Methanol : CH_2Cl_2, 1 : 10 v/v); $[\alpha]_D^{25}$: + 121.8 (*c* 0.43, $CHCl_3$); ^{1}H NMR (400 MHz, $CDCl_3$) for *α*-anomer: δ 6.09 (d, $J_{H-1,H-2}$ = 3.6 Hz, 1H, H-1), 5.69 (d, $J_{H-2,NH}$ = 9.0 Hz, 1H, NH), 5.18 (dd, $J_{H-2,H-3}$ = 11.1 Hz, $J_{H-3,H-4}$ = 9.2 Hz, 1H, H-3), 4.36 (ddd, $J_{H-2,H-3}$ = 11.1 Hz, $J_{H-2,NH}$ = 9.1 Hz, $J_{H-1,H-2}$ = 3.6 Hz, 1H, H-2), 4.29 (dd, $J_{H-6a,H-6b}$ = 12.2 Hz, $J_{H-5,H-6a}$ = 2.5 Hz, 1H, H-6a), 4.25 (dd, $J_{H-5,H-6b}$ = 3.9 Hz, $J_{H-6a,H-6b}$ = 12.2 Hz, 1H, H-6b), 3.82 (ddd, $J_{H-5,H-6a}$ = 2.6 Hz, $J_{H-5,H-6b}$ = 3.8 Hz, $J_{H-4,H-5}$ = 10.0 Hz, 1H, H -5), 3.46 (s, 3H, OMe), 3.42 (dd, $J_{H-3,H-4}$

= 9.4 Hz, $J_{H-4,H-5}$ = 10.0 Hz, 1H, H-4), 2.15 (s, 3H, Ac), 2.11 (s, 3H, Ac), 2.10 (s, 3H, Ac), 1.92 (s, 3H, Ac); ^{13}C NMR (100 MHz, $CDCl_3$): δ 171.6 (CO), 170.5 (CO), 170.0 (CO), 168.7 (CO), 90.7 (C-1), 76.8 (C-4), 73.1 (C-3), 70.8 (C-5), 62.2 (C-6), 60.7 (OMe), 51.2 (C-2), 22.9 (Ac), 20.88 (Ac), 20.8 (Ac), 20.7 (Ac); Selective ^{1}H NMR (400 MHz, $CDCl_3$) for β-anomer: δ 5.76 (d, $J_{H-2,NH}$ = 9.4 Hz, 1H, NH), 5.60 (d, $J_{H-1,H-2}$ = 8.7 Hz, 1H, H-1), 5.00 (dd, $J_{H-2,H-3}$ = 10.7 Hz, $J_{H-3,H-4}$ = 9.0 Hz, 1H, H-3), 3.77 (ddd, $J_{H-4,H-5}$ = 10.1 Hz, $J_{H-5,H-6b}$ = 4.6 Hz, $J_{H-5,H-6a}$ = 2.2 Hz, 1H, H-5); ESI HRMS (m/z): $[M + Na]^+$ calcd. for $C_{15}H_{23}NO_9Na$, 384.1265; found, 384.1262.

Benzyl 2-acetamido-3,6-di-O-acetyl-4-chloro-2,4-dideoxy-α-D-glucopyranoside (**20**): To a solution of compound **18** (30 mg, 0.08 mmol) in anhydrous CH_2Cl_2 (1 mL), was added SO_2Cl_2 (15 μl) and one drop of pyridine at room temperature, and the mixture was heated to 40°C overnight. The reaction mixture was evaporated to dryness under reduced pressure. Anhydrous DMF (1 mL) was added, followed by tetra-*n*-butylammonium chloride (~5. equiv.). After stirring for 15h, the reaction mixture was concentrated under reduced pressure. The crude mixture was purified by column chromatography on silica gel using a gradient of 5→10 % EtOAc–CH_2Cl_2 as the eluent to yield compound **20** (27 mg, 86.5% yield). R_f = 0.53 (EtOAc : CH_2Cl_2, 2 : 8 v/v); ^{1}H NMR (400 MHz, $CDCl_3$): δ 7.45 – 7.31 (m, 5H, Ph), 5.70 (d, $J_{H-2,NH}$ = 9.5 Hz, 1H, NH), 5.28 (dd, $J_{H-2,H-3}$ = 10.2 Hz, $J_{H-3,H-4}$ = 10.2 Hz, 1H, H-3), 4.97 (d, $J_{H-1,H-2}$ = 3.6 Hz, 1H, H-1), 4.75 (d, J = 11.9 Hz, 1H, Bn), 4.55 (d, J = 11.9 Hz, 1H, Bn), 4.44 – 4.35 (m, 2H, H-6a + H-6b), 4.34 – 4.26 (ddd, $J_{H-1,H-2}$ = 3.7 Hz, $J_{H-2,NH}$ = 9.5 Hz, $J_{H-2,H-3}$ = 10.5 Hz, 1H, H-2), 4.10 – 4.03 (m, $J_{H-5,H-6a}$ = 2.3 Hz, $J_{H-5,H-6b}$ = 3.3 Hz, $J_{H-4,H-5}$ = 10.6 Hz, 1H, H-5), 3.97 (dd, $J_{H-3,H-4}$ = 10.2 Hz, $J_{H-4,H-5}$ = 10.2 Hz, 1H, H-4),

2.14 (s, 3H, Ac), 2.11 (s, 3H, Ac), 1.91 (s, 3H, Ac); ^{13}C NMR (100 MHz, $CDCl_3$): δ 171.1 (CO), 170.5 (CO), 170.0 (CO), 136.4 (Ph), 128.7 (Ph), 128.5 (Ph), 128.3 (Ph), 96.7 (C-1), 72.9 (C-3), 70.4 (Bn), 70.3 (C-5), 62.6 (C-6), 55.2 (C-4), 52.6 (C-2), 23.1 (Ac), 20.8 (Ac), 20.7 (Ac); ESI HRMS (m/z): $[M + H]^+$ calcd. for $C_{19}H_{25}ClNO_7$, 414.1314; found, 414.1306.

2-Acetamido-3,6-di-O-acetyl-4-chloro-2,4-dideoxy-α/β-D-glucopyranose (**21**): Compound **18** (90 mg, 0.15 mmol) was hydrogenated in a mixture of methanol (5 mL), CH_2Cl_2 (1.0 mL) and water (4 drops) with 10% Pd-charcoal (80 mg) as above. After stirring overnight, the catalyst was removed by filtration and the crude mixture was purified by column chromatography on silica gel using a gradient of 5→10% EtOAc–CH_2Cl_2 as the eluent to yield compound **20** as an anomeric mixture (α/β, 80/20, 69 mg, 81% yield). R_f = 0.76 (EtOAc : CH_2Cl_2, 2 : 8 v/v); ^{1}H NMR (400 MHz, $CDCl_3$) for the α-anomer: δ 6.02 (d, $J_{NH,H\text{-}2}$ = 9.4 Hz, 1H, NH), 5.34 (dd, $J_{H\text{-}2,H\text{-}3}$ = 10.4 Hz, $J_{H\text{-}3,H\text{-}4}$ = 10.4 Hz, 1H, H-3), 5.28 (dd, $J_{H\text{-}1,H\text{-}2}$ = 3.2 Hz, $J_{H\text{-}1,OH}$ = 3.2 Hz, 1H, H-1), 4.48 (dd, $J_{H\text{-}5,H\text{-}6a}$ = 2.1 Hz, $J_{H\text{-}6a,H\text{-}6b}$ = 12.2 Hz, 1H, H-6a), 4.39 (br, 1H, OH), 4.35 (dd, $J_{H\text{-}5,H\text{-}6b}$ = 4.0, $J_{H\text{-}6a,H\text{-}6b}$ = 12.2 Hz, 1H, H-6b), 4.31 – 4.20 (m, 1H, H-5 + H-2), 3.97 (dd, $J_{H\text{-}3,H\text{-}4}$ = 10.3 Hz, $J_{H\text{-}4,H\text{-}5}$ = 10.3 Hz, 1H, H-4), 2.13 (s, 6H, 2 × Ac), 1.99 (s, 3H, Ac); ^{13}C NMR (101 MHz, $CDCl_3$) for the α-anomer: δ 171.2 (CO), 170.8 (CO), 170.6 (CO), 91.7 (C-1), 72.6 (C-3), 69.9 (C-5), 62.8 (C-6), 55.3 (C-4), 53.1 (C-2), 23.1 (Ac), 20.8 (Ac), 20.7 (Ac); ^{1}H NMR (400 MHz, $CDCl_3$) for the β-anomer: δ 6.34 (d, $J_{NH,H\text{-}2}$ = 7.0 Hz, 1H, NH), 5.47 (d, $J_{H\text{-}1,OH}$ = 7.5 Hz, OH), 5.05 (dd, $J_{H\text{-}2,H\text{-}3}$ = 9.9 Hz, $J_{H\text{-}3,H\text{-}4}$ = 10.6 Hz, 1H, H-3), 4.67 (dd, $J_{H\text{-}1,H\text{-}2}$ = 7.6 Hz, $J_{H\text{-}1,OH}$ = 7.6 Hz, 1H, H-1), 4.49 (dd, $J_{H\text{-}5,H\text{-}6a}$ = 2.0 Hz, $J_{H\text{-}6a,H\text{-}6b}$ = 12.2 Hz, 1H, H-6a), 4.36 (overlapped, 1H, H-6b), 3.92 (dd, $J_{H\text{-}3,H\text{-}4}$ = 10.1 Hz, $J_{H\text{-}4,H\text{-}5}$ = 10.1 Hz, 1H, H-4), 3.75 (ddd, 1H, $J_{H\text{-}5,H\text{-}6a}$ = 2.0 Hz, $J_{H\text{-}5,H\text{-}6b}$ = 4.6 Hz, $J_{H\text{-}4,H\text{-}}$

$_{5}$ = 10.4 Hz, H-5), 2.18 (s, 3H, Ac), 2.13 (overlapped, 3H, Ac), 2.03 (s, 3H, Ac); Selective ^{13}C NMR (100 MHz, $CDCl_3$) for the *β*-anomer: *δ* 97.7 (C-1), 74.5 (C-3), 74.1 (C-5), 62.8 (C-6), 57.9 (C-4), 55.0 (C-2); ESI HRMS (m/z): $[M + Na]^+$ calcd. for $C_{12}H_{18}ClNO_7Na$, 346.0664; found, 346.0657.
